# *Prunus dulcis* response to novel defense elicitor peptides and control of *Xylella fastidiosa* infections

**DOI:** 10.1007/s00299-024-03276-x

**Published:** 2024-07-08

**Authors:** Luis Moll, Núria Giralt, Marta Planas, Lidia Feliu, Emilio Montesinos, Anna Bonaterra, Esther Badosa

**Affiliations:** 1 Laboratory of Plant Pathology, Institute of Food and Agricultural Technology-CIDSAV, University of Girona, Campus Montilivi, 17003 Girona, Spain; 2 LIPPSO, Department of Chemistry, University of Girona, Campus Montilivi, 17003 Girona, Spain

**Keywords:** Plant defense elicitor, Almond, *Prunus dulcis*, **flg22**, Endotherapy, Plant response, *Xylella fastidiosa*

## Abstract

**Key message:**

New defense elicitor peptides have been identified which control *Xylella fastidiosa* infections in almond.

**Abstract:**

*Xylella fastidiosa* is a plant pathogenic bacterium that has been introduced in the European Union (EU), threatening the agricultural economy of relevant Mediterranean crops such as almond (*Prunus dulcis*). Plant defense elicitor peptides would be promising to manage diseases such as almond leaf scorch, but their effect on the host has not been fully studied. In this work, the response of almond plants to the defense elicitor peptide **flg22-NH**_**2**_ was studied in depth using RNA-seq, confirming the activation of the salicylic acid and abscisic acid pathways. Marker genes related to the response triggered by **flg22-NH**_**2**_ were used to study the effect of the application strategy of the peptide on almond plants and to depict its time course. The application of **flg22-NH**_**2**_ by endotherapy triggered the highest number of upregulated genes, especially at 6 h after the treatment. A library of peptides that includes **BP100-flg15**, **HpaG23**, **FV7**, **RIJK2**, **PIP-1, Pep13**, **BP16-Pep13**, **flg15-BP100** and **BP16** triggered a stronger defense response in almond plants than **flg22-NH**_**2.**_ The best candidate, **FV7**, when applied by endotherapy on almond plants inoculated with *X. fastidiosa,* significantly reduced levels of the pathogen and decreased disease symptoms. Therefore, these novel plant defense elicitors are suitable candidates to manage diseases caused by *X. fastidiosa*, in particular almond leaf scorch.

**Supplementary Information:**

The online version contains supplementary material available at 10.1007/s00299-024-03276-x.

## Introduction

*Xylella fastidiosa* is a Gram-negative, aerobic, xylem-limited bacterium responsible for several plant diseases such as Pierce’s disease, citrus variegated chlorosis, and almond leaf scorch (ALS), and is a great threat to the agriculture worldwide (Wells et al. 1987; Alston et al. 2013; Purcell 2013; Rapicavoli et al. 2018a). This pathogen has been detected in the EU territories and is currently spreading through the Mediterranean region, threatening the agricultural economy of global producers of olives, citrus, almonds, and grapes (Strona et al. 2017; Saponari et al. 2019; Gibin et al. 2023). In fact, the potential economic losses associated with the full spread of *X. fastidiosa* in the EU amount to an average of 5.5 billion euros per year and has been ranked as a priority quarantine pathogen in the area (Sánchez et al. 2019). One of the main affected crops in the EU is almond (*Prunus dulcis*), with Spain being one of its main producers worldwide, with a production of almost 250.000 tons in 2022 (FAO 2024). *X. fastidiosa* causes ALS in almond and consists of an initial leaf scorching, followed by a general decline of the trees, which leads to a reduction of their health and productivity between 20 and 40%, and may eventually result in the death of the tree (Baró et al. 2021; Marco-Noales et al. 2021). At the present, most of the measures adopted to manage diseases caused by *X. fastidiosa* are focused on eradication, limiting the spread of the bacterium by means of vector control, and replacing susceptible varieties for tolerant ones (Bragard et al. 2019; Carluccio et al. 2023; Avosani et al. 2024; Cornara et al. 2024). Other methods that are under study rely on the reduction of the pathogen population in infected plants using the endophyte *Paraburkholderia phytofirmans* (Baccari et al. 2019; Lindow et al. 2023)*,* avirulent *X. fastidiosa* strains (Hao et al. 2017), lytic phages, and chemical compounds such as copper (II) and citric acid fertilizers (Amanifar et al. 2016; Scortichini et al. 2018; Ge et al. 2020). Although considerable research has been performed, there is still no strategy to protect and completely cure infected plants (Burbank 2022).

A method to protect plants from pathogen infection or reduce disease severity involves the activation of the plant immune system, known as induced resistance (Reglinski et al. 2023). The plant immune response is mediated by a complex network of signals that include several phytohormones [salicylic acid (SA), jasmonic acid (JA), ethylene (Et) and abscisic acid (ABA)] (Li et al. 2019; Ali and Baek 2020; Lefevere et al. 2020). Once the plant defense system has been activated, the plant enters into a primed state through an accumulation of pathogenesis related (PR) and signal transduction proteins (kinases or transcription factors) (Conrath 2011; Martinez-Medina et al. 2016; Hilker et al. 2016). Primed plants present a better performance when infection occurs compared to non-primed plants (van Hulten et al. 2006; Martinez-Medina et al. 2016).

Defense elicitor peptides, either synthetic or of natural origin, are considered as suitable candidates for plant disease control (Montesinos 2023). On the one hand, short plant endogenous elicitor peptides PROPEPs and Peps have been reported to induce plant defenses (Bartels and Boller 2015; Ruiz et al. 2018), and, in a recent study, the topical application of Peps (**PpPep1** and **PpPep2**) protected *Prunus persica* plants against *Xanthomonas arboricola* pv. pruni infection (Foix et al. 2021). On the other hand, several synthetic or microbial-derived peptides induce plant defenses, thus acting as microbial or pathogen-associated molecular patterns (MAMPS or PAMPs). Examples of these peptides are **flg15** and **flg22-OH** (Felix et al. 1999), the linear lipopeptide **BP473** (Oliveras et al. 2021), the bifunctional peptide **BP178** (Badosa et al. 2013; Montesinos et al. 2021), **HpaG23** (Kim et al. 2004), the hexapeptide **PIP-1** (Miyashita et al. 2011), and the peptide derived from citrus **MaSAMP** (Huang et al. 2021). Interestingly, some of these peptides also have a direct effect onto the pathogen such as the multifunctional peptide **MaSAMP,** which activates the plant defense responses in *Nicotiana benthamiana* and *Solanum lycopersicum* and displays antibacterial activity against the endophytic bacteria *Candidatus* Liberibacter (Huang et al. 2021). Another interesting peptide is **BP178**, identified in our group from a collection of peptide conjugates including two antimicrobial sequences (Badosa et al. 2013)**. BP178** exhibits high antibacterial activity against *X. fastidiosa*, *Pseudomonas syringae* pv. *tomato,* and* X. campestris* pv. *campestris* among others, together with plant defense elicitor properties in *N. benthamiana, S. lycopersicum,* and *Prunus dulcis* (Badosa et al. 2013; Montesinos et al. 2021; Moll et al. 2022). We also reported multifunctional peptides obtained from the conjugation of a plant defense elicitor and an antimicrobial peptide such as **flg15-BP475** and **flg15**-**BP16** (Oliveras et al. 2022; Caravaca-Fuentes et al. 2021).

The most studied plant defense elicitor peptide is **flg22-OH**, a 22 amino acid sequence of the N-terminus of flagellin of *Pseudomonas aeruginosa* (Felix et al. 1999; Zipfel et al. 2004; Sun et al. 2013; Liu et al. 2015; Czékus et al. 2023). Plant species such as *A. thaliana, S. lycopersicum,* and *N. tabacum* respond to **flg22-OH** (Zipfel et al. 2004; Sun et al. 2013; Liu et al. 2015; Montesinos et al. 2021; Czékus et al. 2023), whereas other species like *Actinidia arguta* do not have **flg22-OH** receptors but recognize other epitopes of flagellin such as **flgII-28** or **CD2-1** (Trdá et al. 2014; Veluchamy et al. 2014; Ciarroni et al. 2018; Murakami et al. 2022). Thus, plant responses to a given elicitor peptide may differ among species and it is necessary to test elicitor peptides directly in the crop plant species of interest.

Therefore, the aim of the present work was to identify new peptides with plant defense elicitor activity, which in turn would be able to protect almond from *X. fastidiosa* infections. Specific objectives were (i) to analyze in depth the differential expression of genes after treatment of *P. dulcis* with **flg22-NH**_**2**_ to select suitable markers of plant defense response to peptides, (ii) to evaluate different strategies for the peptide application to the plant, and to test a group of new peptides, and finally (iii) to test the effect of the treatment with the best peptide on the population levels of *X. fastidiosa* and ALS severity on almond plants under greenhouse conditions.

## Materials and methods

### Selection, design, and synthesis of peptides

With the aim of identifying peptides with plant defense elicitor activity on *P. dulcis*, 25 sequences were selected (Table 1). Peptides **flg22-NH**_**2**_, **flg22-OH**, **flg15, elf18-NH2, elf18-OH, csp15, HpaG23, Pep13, PIP-1,** and **BP13** were chosen for their previously described activity as plant defense elicitors in other model plants (Nürnberger et al. 1994; Felix et al. 1999; Felix and Boller 2003; Kim et al. 2004; Kunze et al. 2004; Miyashita et al. 2011; Badosa et al. 2017). Peptides **BP100**, **BP16**, **KSLW**, **1036**, **RIJK2,** and **FV7** were selected because they display high antibacterial or antibiofilm activity against Gram-negative bacteria, being good candidates to be tested as plant defense elicitors (Na et al. 2007; Badosa et al. 2007; De La Fuente-Núñez et al. 2012, 2015; Xu et al. 2014; Moll et al. 2021). Peptide conjugates **BP100-flg15**, **flg15-BP100**, **BP16-Pep13**, **Pep13-BP16**, **flg15-BP16,** and **BP16-flg15,** incorporating a peptide elicitor (**flg15**, **Pep13**) and an antimicrobial peptide (**BP100**, **BP16**), were also included in this study (Caravaca-Fuentes et al. 2021; Oliveras et al. 2022). Peptide **flg15-BP16** was previously identified in our group as a multifunctional peptide with the ability to induce defense responses in tomato plants and to reduce the severity of fire blight in pear plants (Caravaca-Fuentes et al. 2021). In addition, we also designed new peptide conjugates resulting from the combination of two antimicrobial and/or antibiofilm sequences, **KSLW-BP100**, **BP16-KSLW** and **KSLW-FV7**.

**Table 1:** Codes and sequences of the peptides used in this study

Code	Sequence^a^	Reference^b^
flg22-NH_2_	QRLSTGSRINSAKDDAAGLQIA-NH_2_	This study
flg22-OH	QRLSTGSRINSAKDDAAGLQIA-OH	Felix et al. 1999
flg15	RINSAKDDAAGLQIA-OH	
elf18-NH_2_	Ac-SKEKFERTKPHVNVGTIG-NH_2_	This study
elf18-OH	Ac-SKEKFERTKPHVNVGTIG-OH	Kunze et al. 2004
csp15	VKWFNAEKGFGFITP-OH	Felix and Boller 2003
HpaG23	NQGISEKQLDQLLTQLIMALLQQ-OH	Kim et al. 2004
Pep13	VWNQPVRGFKVYE-OH	Nürnberger et al. 1994
PIP-1	YGIHTH-NH_2_	Miyashita et al. 2011
BP13	FKLFKKILKVL-NH_2_	Badosa et al. 2007
BP100	KKLFKKILKYL-NH_2_	
BP16	KKLFKKILKKL-NH_2_	
KSLW	KKVVFWVKFK-NH_2_	Na et al. 2007
1036	VQFRIRVRIVIRK-NH_2_	De La Fuente-Núñez et al. 2012
RIJK2	rivwvrirrwfv-NH_2_	De La Fuente-Núñez et al. 2015
FV7	FRIRVRV-NH_2_	Xu et al. 2014
BP100-flg15	KKLFKKILKYL-RINSAKDDAAGLQIA-OH	Oliveras et al. 2022
flg15-BP100	RINSAKDDAAGLQIA-KKLFKKILKYL-NH_2_	
BP16-Pep13	KKLFKKILKKL-VWNQPVRGFKVYE-OH	
Pep13-BP16	VWNQPVRGFKVYE-KKLFKKILKKL-NH_2_	
flg15-BP16	RINSAKDDAAGLQIA-KKLFKKILKKL-NH_2_	Caravaca-Fuentes et al. 2021
BP16-flg15	KKLFKKILKKL-RINSAKDDAAGLQIA-OH	
KSLW-BP100	KKVVFWVKFK-KKLFKKILKYL-NH_2_	This study
BP16-KSLW	KKLFKKILKKL-KKVVFWVKFK-NH_2_	
KSLW-FV7	KKVVFWVKFK-FRIRVRV-NH_2_	

^a^Lowercase amino acid stands for the corresponding D-isomer

^b^Each reference belongs to the indicated peptide and the ones below until a new reference is indicated

Peptides were synthesized manually on solid phase using a standard 9-fluorenylmethoxycarbonyl (Fmoc)/*tert-*butyl (*t*Bu) strategy. Fmoc-Rink-ChemMatrix (0.69 mmol/g), PAC-ChemMatrix (0.22 mmol/g), or Fmoc-Rink-MBHA (0.71 mmol/g) resins were used as solid support. Fmoc-Rink-ChemMatrix and PAC-ChemMatrix resins were selected for the synthesis of peptides containing more than 14 residues. The PAC-ChemMatrix resin was employed to prepare C-terminal carboxylic acid peptides, whereas the Fmoc-Rink-ChemMatrix and the Fmoc-Rink-MBHA resins served for C-terminal peptide amides. Peptide elongation was carried out through sequential steps of Fmoc removal and coupling of the corresponding amino acid as previously described (Caravaca-Fuentes et al. 2021; Oliveras et al. 2021). Once the peptide sequence was completed, each resulting peptidyl resin was treated with trifluoroacetic acid (TFA)/H_2_O/triisopropylsilane (TIS) (95:2.5:2.5). Peptidyl resins that contained tryptophan and/or arginine were treated with TFA/H_2_O/TIS/thioanisole/1,2-ethanditiol/phenol (81.5:5:1:5:2.5:5). Following TFA evaporation and diethyl ether extraction, the crude peptides were purified by reverse-phase column chromatography, lyophilized, analyzed by HLPC, and characterized by mass spectrometry. All peptides were obtained in excellent HLPC purities (≥ 94%) and their identity was confirmed by mass spectrometry (Supplementary Table S1).

## Plants and greenhouse conditions

One-year-old almond plants (*P. dulcis*) from the cv. Avijor provided by Agromillora S. L. U. (Spain) were used for the experiments. All plants were maintained in 0.8 L pots (sphagnum peat with wood fiber (10%), calcium carbonate (9 g/liter), NPK fertilizer (1 kg/m^3^), and microelements) in an environmentally controlled greenhouse. The photoperiod consisted of 16 h of light at 25 ± 2 °C (day) and 8 h of darkness at 18 ± 2 °C (night). Prior and during the experiments, plants were watered to saturation every 3 days, and fertilized with a 200 ppm solution of NPK (20:10:20) once a week. In addition, throughout the experiments, standard treatments with insecticide and acaricide were performed to avoid the presence of insect vectors or pests, except in plants used for transcriptomic analyses. Infected plants were cultivated in a Biosafety level II + quarantine greenhouse authorized by the Plant Health Services, according to EPPO-recommended containment conditions (EPPO 2006) and maintained taking into account the consideration of *X. fastidiosa* as a quarantine pathogen in the EU (EC 2019).

## Peptide application systems in almond plants and RNA extraction for gene expression analysis

The plant defense elicitor activity of **flg22-NH**_**2**_ was determined on almond plants and, then, this peptide was used as a reference in additional experiments. Additionally, the previously mentioned peptides were included in the screening of plant defense elicitor experiment. Before use, lyophilized peptides were solubilized in sterile Milli-Q water to a stock concentration of 20 mM. Depending on the experiment, the peptide was applied through: (i) endotherapy followed by a spray treatment, (ii) endotherapy, (iii) spray, or (iv) infiltration into the leaves. Endotherapy treatments consisted of an injection of 10 µL of the peptide at 20 mM for each plant using a high precision microinjector (NanoJet, Chemyx, Stafford, USA) provided with a Hamilton 250 μL syringe with a thin needle with bevel tip (Fisher Scientific, New Hampshire, USA). The application was performed 20 cm below the most apical region of the plant. The needle end was introduced until approximately one-half of the plant stem diameter to reach the vascular system (Moll et al. 2022). Leaf samples were gathered above the application point. Spray treatments consisted of the application of 2 mL of the peptide at 125 µM on the adaxial and abaxial leaf surfaces using an airbrush until near runoff (Herkules, Nuair, Robassomero, Italy) (Montesinos et al. 2021). The treatment was applied to all of the leaves of the plant and samples were gathered at 15 cm below the most apical region of the plant. Infiltration into the leaves was done by performing a small incision with a needle into the abaxial side of the leaves and infiltrating 50 μL of the peptide at 1 μM into the mesophyll using a syringe (Giolai et al. 2019). Treated leaves were marked to be sampled later. Plants treated with water were used as control in all of the experiments.

For the RNA-seq experiments, endotherapy followed by a spray treatment was applied to four biological replicates of five plants. For each treated and not treated plant, a total of four leaves were sampled which resulted in a pool of 20 leaves for each biological replicate. Sampling was performed at 6 and 24 h post-treatment (hpt). For the RT-qPCR experiments, endotherapy, spray, or infiltration into the leaves was applied to three biological replicates of three plants. For each treated and not treated plant, a total of 4 leaves were sampled which resulted in a pool of 12 leaves for each biological replicate. Sampling was performed at 6 hpt for the other experiments except for the gene expression kinetic in which the samples of the treated and not treated plants were gathered at 1, 3, 6, and 12 hpt.

Once sampled, leaves were immediately frozen in liquid nitrogen and finely ground. They were transferred to tubes with two glass beads and homogenized with a Tissue Lyser II (Qiagen, Hilden, Germany) at a frequency of 30 Hz for 1 min. Homogenized samples were kept at −70 °C until RNA extraction. RNA was extracted from 100 mg of the ground leaf material from each biological replicate using the PureLink™ Plant RNA Reagent (Invitrogen Life Technologies, Carlsbad, CA, USA), and the remaining DNA was digested with the TURBO DNA-*free*™ Kit (Invitrogen Life Technologies, Carlsbad, CA, USA), following the manufacturer’s instructions. RNA concentration was estimated through absorbance at 260 nm and RNA quality was assessed with the 260/280 and the 260/230 ratios using a NanoDrop ND1000 spectrophotometer (Nanodrop Technologies, Wilmington, DE, USA).

## RNA-seq analysis

The effect of the peptide **flg22-NH**_**2**_ on *P. dulcis* cv. Avijor transcriptome response at 6 and 24 hpt was assessed through RNA-seq. RNA samples were stabilized at room temperature using the RNA Transport kit (Omega Bio-tek, Norcross, GA, USA) and sent to Sequentia Biotech (Barcelona, Spain) for RNA sequencing. RNA “TruSeq Stranded mRNA Sample Prep kit” (Illumina, San Diego, CA) was used for library preparation following the manufacturer’s instructions, starting with 1–2 µg of good-quality RNA (RIN > 7) as input. The RNA was fragmented 3 min at 94 °C and every purification step was performed using 0.81X Agencourt AMPure XP beads. Both RNA samples and final libraries were quantified using the Qubit 2.0 Fluorometer (Invitrogen, Carlsbad, CA, USA) and quality tested by Agilent 2100 Bioanalyzer RNA Nano assay (Agilent technologies, Santa Clara, CA, USA). Libraries were then processed with Illumina cBot for cluster generation on the flowcell, following the manufacturer’s instructions and sequenced on paired-end (2 × 150 bp, 30 M reads per sample) at the multiplexing level requested on NovaSeq6000 (Illumina, San Diego, CA, USA). The CASAVA 1.8.2 version of the Illumina pipeline was used to process raw data for both format conversion and de-multiplexing.

Raw sequence files were first subjected to quality control analysis using FastQC v0.10.1 (https://www.bioinformatics.babraham.ac.uk/projects/fastqc/) before trimming and removal of adapters with BBDuk (https://jgi.doe.gov/data-and-tools/bbtools/), setting a minimum base quality of 25 and a minimum read length of 35 bp. Reads were then mapped against the *P. dulcis* genome (Sánchez-Pérez et al. 2019) with STAR v2.6 (Dobin et al. 2013). FeatureCounts v1.6.1 (Liao et al. 2014) was then used to obtain raw expression counts for each annotated gene using only uniquely mapping reads (MAPQ ≥ 30). The differential gene expression analysis was conducted with the R package edgeR (Robinson et al. 2010) using the Trimmed mean of M-values (TMM) normalization method and considering as significant the genes with a false discovery rate (FDR) ≤ 0.05. Fragments per kilobase million (FPKM) were obtained with edgeR. Gene ontology enrichment analysis (GOEA) was performed using in-house scripts based on the AgriGO publication (Tian et al. 2017).

Differently expressed genes (DEGs) with an FDR < 10^–2^ and a log_2_ fold change (FC) ≥|1| were selected. The information of the selected genes was obtained from databases of *P. dulcis* genes (GenBank; https://www.ncbi.nlm.nih.gov/genbank/ and Uniprot; https://www.uniprot.org/). The closest gene homologs of *P. dulcis* genes were found in *A. thaliana* and functional information was complemented (when available). Functional information was obtained from several databases, such as GenBank, Uniprot and The Arabidopsis Information Resource (TAIR, https://www.arabidopsis.org/). Using this information, genes were classified into terms (defense pathways such as SA, ABA, JA and Et and non-defense such as development, metabolism, and other when possible).

RNA-seq data have been deposited in the GEO-NCBI repository with the code number GSE259385.

The RNA-seq data were analyzed to assess the general effect of **flg22-NH**_**2**_ treatment onto the almond transcriptome. Relevant DEGs identified in almond of the different pathways were portrayed onto the general plant defense response representation using previous reported studies.

## Quantitative real-time PCR analyses

According to the results of the RNA-seq analysis, a total of 15 DEGs were selected among several defense and non-defense-related pathways. For each of these genes, primers were designed using Primer-BLAST (NCBI, USA) (Supplementary Table S2). First-strand complementary DNA (cDNA) was generated from leaf RNA using reverse transcriptase (RT) (high-capacity cDNA reverse transcription kit; Applied Biosystems, Foster City, CA, USA) according to the manufacturer’s manual. cDNA was amplified through polymerase chain reaction (PCR) using the following conditions: 5 min at 95 °C, 35 cycles of 30 s at 95 °C, 30 s at 60 °C and 30 s at 72 °C; and 3 min at 72 °C. Each reaction consisted of 13.8 µL of DEPC-treated water, 2 µL of PCR 10 × buffer, 0.8 µL of MgCl_2_ at 50 mM, 0.4 µL of dNTPs at 10 mM, 0.4 µL of forward primer at 10 µM, 0.4 µL of reverse primer at 10 µM, 0.2 of µL of Taq polymerase at 10 U/µL, and 2 µL of cDNA at 1.6 µg/µL. Primers were purchased from Merck (Darmstadt, Germany) and reagents were purchased from Invitrogen (Waltham, Massachusetts, USA). All PCR products were sent to Macrogen for sequencing (Amsterdam, The Netherlands).

PCR products were cloned using the pSpark DNA cloning system (Canvax, Córdoba, Spain) following the manufacturer’s instructions and were used to transform *Escherichia coli* DH5α. Plasmids were purified using the QIAprep® Spin Miniprep kit (Qiagen Ibera, S.L.; Madrid, Spain) according to the manufacturer’s manual and quantified as a copy number. They were used for quantitative real-time PCR analyses (qPCR) primer optimization (7500 Fast Real-Time PCR System, Applied Biosystems, Foster City, CA, USA). Tested final forward and reverse primer concentrations corresponded to 100 nM, 300 nM, and 600 nM and all its combinations. The qPCR reaction conditions were as follows: 10 min at 95 °C; 40 cycles of 15 s at 95 °C, and 1 min at 60 °C; and a final melting curve program of 60–95 °C with a heating rate of 0.5 °C/s. qPCRs were performed in a 96-well plate and each reaction consisted of 6 µL of DEPC-treated water, 10 µL of SYBR™ Green PCR Master Mix (Applied Biosystems), 1 µL of forward and reverse primer at 2, 6, or 12 µM, and 2 µL of the sample. Technical duplicates of each sample were performed. Decimal dilutions of the plasmids from 10^8^ to 10^2^ copies were prepared and calibration curves were obtained. RT-qPCR was performed in newly extracted RNA to validate the RNA-seq results using the conditions described above. The efficiency was calculated to check that it was similar between the selected genes (Supplementary Table S2). Relative quantification of gene expression was done using the ΔΔ*C*_*T*_ method (Livak and Schmittgen 2001). The transcription elongator factor 2 (*TEF2*; Prudu.04G124200) and Ubiquitin 10 (*UBQ*; Prudu.04G183800) (Foix et al. 2021) were tested as an endogenous reference gene to evaluate and validate the most appropriate endogenous gene to normalize gene expression data according to the method described by Silver et al. (2006).

## Screening of plant defense elicitor peptides on almond plants

A total of 25 peptides (Table 1) were tested as plant defense elicitors on almond plants. Peptide **flg22-NH**_**2**_ was used as a reference. Peptides were applied through endotherapy at 20 mM and sampling was carried out at 6 hpt. RNA extraction, RT-qPCR, and relative quantification of gene expression using the ΔΔ*C*_*T*_ method was performed as described above for 11 of the 15 selected genes (Supplementary Table S2). Genes were considered to be upregulated when they showed significant differences between their respective NTC (*p* < 0.05) and their fold change was higher than 1.5. The intensity of expression was calculated for each peptide as a numeric value that corresponds to the sum of the intensity of upregulation of each gene, which is 0 when < 1.5-fold change, 1 when 1.5–3.5, and 2 when > 3.5.

## *X. fastidiosa* strain and growth conditions

*X. fastidiosa* subs. *fastidiosa* IVIA 5387.2 (*Xff*) (Moralejo et al. 2019), isolated from almond trees in Mallorca (Spain) and kindly provided by the Instituto Valenciano de Investigaciones Agrarias (IVIA), was used in the plant experiments. The strain was stored in Pierce disease broth (PD2, Davis 1980) supplemented with glycerol (30%) and maintained at −80 °C. When needed, aliquots were cultured in buffered charcoal yeast extract agar plates (BCYE, Wells et al. 1981) and grown at 28 °C for two passages of 7 days each. A cell suspension was prepared in PD3 (Davis et al. 1981) and adjusted to 10^8^ CFU/mL (OD_600_ $\cong$ 0.3), confirmed by plate counting as previously described (Baró et al. 2021).

## Effect of selected peptides on the population levels of *X. fastidiosa,* ALS severity, and selected leaf physiological parameters in almond plants

Peptides **flg22-NH**_**2**_, **FV7,** and **1036** were evaluated for their effect on the population levels of *Xff*, ALS severity, and selected leaf physiological parameters in inoculated almond plants of cultivar Avijor compared to a not treated control (NTC) and not inoculated plants. **FV7** was selected since it presented high plant defense elicitor activity in almond, did not have bactericidal activity against *Xff,* and consisted of a short amino acid sequence, facilitating its synthesis (Moll et al. 2021). **flg22-NH**_**2**_ was chosen for comparative reasons, since it is a widely studied plant defense elicitor in many plant species, and additionally no bactericidal activity was observed using the method described by Moll et al. 2021. **1036** was selected as a peptide with no elicitor activity, although it presented high bactericidal activity against *Xff* (Moll et al. 2021).

Peptides were applied through endotherapy as explained previously in this work and the pathogen was inoculated by microinjection as described previously (Moll et al. 2022). Briefly, the peptide was applied 1 day before *Xff* inoculation and 3 and 7 days post-inoculation (dpi), and each application consisted of three shoots of 10 μL at 20 mM using the high-precision microinjector as shown in Supplementary Fig. 1. NTCs were obtained using water instead of the peptides. The inoculation of *Xff* was performed as described in Baró et al. 2021. Plants were inoculated with three injections of 10 μL of a suspension of *Xff* at 10^8^ CFU/mL, equivalent to a total of 3 × 10^6^ CFU/plant. The injections were performed on the same side of the stem in a section of 3 cm at around 15 cm above the substrate level (Supplementary Fig. 1). Not inoculated controls were included by injecting water instead of the bacterial suspension and the peptide.

*X. fastidiosa* population levels were assessed for all the treatments (not inoculated, NTC, **flg22-NH**_**2**_**, FV7,** and **1036**). The experimental design consisted of three replicates of three plants per each treatment and sampling time (15, 40, 65 and 90 dpi) (180 plants). A second experiment was carried out by only sampling at 40 dpi (45 plants). Samples were collected and the population levels of *X. fastidiosa* cells in sap were analyzed as described in Baró et al. 2021. Briefly, to determine the spread and multiplication of the pathogen from the inoculated area, 16 cm of shoot material was sampled above the inoculation points (upward zone 1; upward zone 2; 8 cm each zone), and below (downward zone; 8 cm) (Supplementary Fig. 1). Sap was obtained from each 8-cm fragment by removing the bark from the stems to mostly retain vascular tissue, cutting the fragment into three parts, and putting them in 2-mL centrifuge tubes with a hole at the bottom. The 2-mL tubes were inserted in 5-mL tubes, and the assembly was centrifuged at 15.800*g* for 25 min. The population levels of *Xff* in sap were analyzed by viability-qPCR (v-qPCR) (Baró et al. 2020b). The sap of three plants was collected in the 5-mL tube and diluted to a final volume of 500 μL of PBS. For v-qPCR, an aliquot of 200 μL was treated with PMAxx to a final concentration of 7.5 μM (VWR, Barcelona, Spain), incubated for 8 min in the dark at room temperature, and photoactivated for 15 min (PMA-Lite™ LED Photolysis Device, Biotium, CA, USA) (Moll et al. 2021). DNA extraction was performed using the GeneJET Genomic DNA purification Kit (Thermo Fisher Scientific) following the manufacturer’s instructions. Finally, a TaqMan-based qPCR was used as described previously (Baró et al. 2020a). The number of viable cells in sap, expressed as log_10_ CFU/mL, was obtained by interpolating C_T_ values from samples of the experiment in a standard curve, CFU versus C_T_ values, and made with sap from a healthy almond plant of cultivar Avijor fortified with known concentrations of *Xff.*

ALS symptoms were also assessed following the severity scale previously described in the literature (Baró et al. 2021). The experimental design consisted of three replicates of three plants per each treatment (Not inoculated, NTC, **flg22-NH**_**2**_, **FV7,** and **1036**) (45 plants). Two independent experiments were performed. Symptom evaluation was performed at 0, 15, 30, 47, 58, 70, 82, and 90 dpi. Additionally, chlorophyll, flavonol, and anthocyanin content were determined by leaf transmittance providing an index which is proportional to the content of each compound within the leaf using the DUALEX sensor (METOS Iberia, Seville, Spain) at the same time stamps (Cerovic et al. 2012; Camino et al. 2021). A not inoculated control was also included since it has been described that *X. fastidiosa* infection alters the previously mentioned parameters (Zarco-Tejada et al. 2018; Pereira et al. 2019; Camino et al. 2021). Briefly, measurements were taken on four leaves above the highest point of inoculation between the center and the margin of the leaves. A total of 12 leaves were pooled from three different plants for each replicate.

## Data analysis

The statistical significance of the effect of the peptides on the expression of the selected genes was determined using REST2009 software between treated and not treated samples (*p* < 0.05) (Qiagen Ibera, S.L., Barcelona, Spain). All data were tested for normality using the Shapiro–Wilk test and for homogeneity of variances using the Levene test. To test the significance between application systems and sampling times, a one-way analysis of variance (ANOVA) was performed. To test the significance of the effect of peptides on *Xff* population levels, ALS symptoms, and leaf physiological parameters (chlorophyll, flavonol and anthocyanin contents) over time, a repeated measures ANOVA was used. In all cases, means were separated according to the Duncan’s test at a *p *value of < 0.05 (IBM SPSS, Statistics, for Windows, Version 25.0 released on 2017 by IBM Corp, Rmonk, NY, USA). The hierarchical clustering using the Euclidean distance for the identification of new plant defense elicitors on almond was performed using the default heatmap() function in RStudio version 2022.07.1 Build 554 (Boston, MA, USA).

## Results

### Analysis of the differential expression of genes after treatment of almond plants with flg22-NH_2_

Sixteen mRNA libraries were sequenced from four replicates of *P. dulcis* “Avijor” treated with **flg22-NH**_**2**_ after 6 and 24 hpt and each corresponding NTC. Each library included approximately between 15 and 19 million raw reads from which, after filtering for high-quality reads, 14–17 million sequences were kept. Reads were assigned to the *P. dulcis* reference genome (Sánchez-Pérez et al. 2019), and between 75 and 77% of them were uniquely mapped to genes (Supplementary Tables S3 and S4). The overall quality of the experiment was assessed using a principal component (PC) analysis. The component PC1 accounted for 88.5% of the total variation in the dataset, which resulted in two clusters corresponding to 6 and 24 hpt, respectively (Supplementary Fig. S2A). Each sampling time was then analyzed independently. In the case of 6 hpt, one of the biological replicates of the NTC was removed because it appeared as an outlier (Supplementary Fig. S2B, C). This modification resulted in two clusters that clearly separated treated from not treated plants at 6 hpt. This separation was not so defined at 24 hpt (Supplementary Fig. S2D). After bioinformatic analysis (Supplementary Fig. S3 and Tables S5 and S6), differentially expressed genes (DEGs) were identified, which after filtering (FDR ≤ 10^–2^ and log_2_ FC ≥|1|) led to 123 upregulated and 46 downregulated genes at 6 hpt, and 39 upregulated and 32 downregulated genes at 24 hpt.

**Fig. 1:**
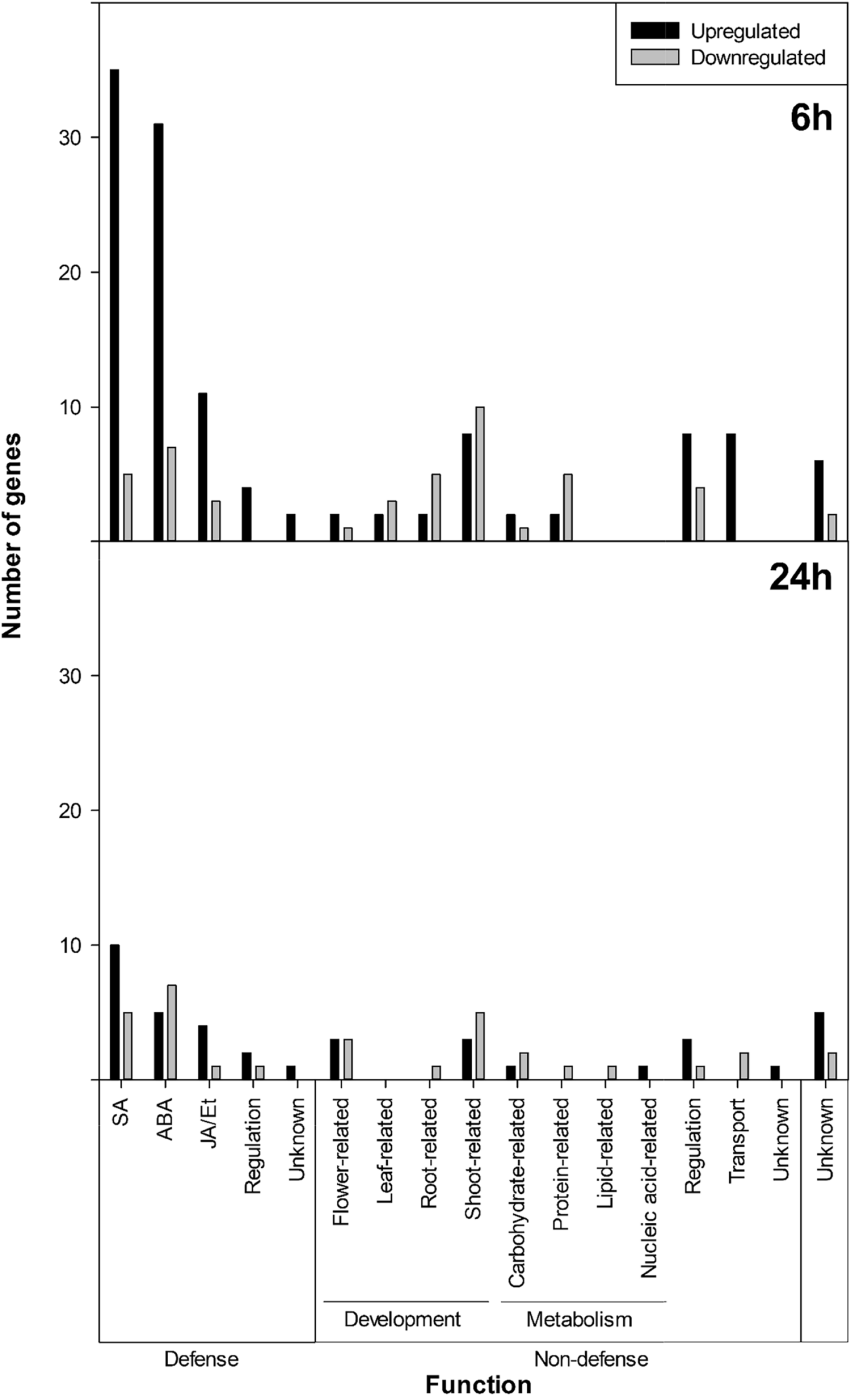
Number of differentially upregulated and downregulated genes in almond plants after **flg22-NH**_**2**_ treatment at 6 and 24 h classified in defense and non-defense pathways

DEGs were assigned to different groups based on functional information and were categorized into defense (SA, ABA, JA/Et) and non-defense (development, metabolism, and others) pathways (Fig. 1). At 6 h after treatment with **flg22-NH**_**2**_**,** the number of transcripts involved in defense functions was higher than at 24 hpt. In particular, 83 genes were upregulated (68%) and 15 downregulated (33%), whereas at 24 hpt, 22 genes were upregulated (56%) and 14 were downregulated (44%).

It is interesting to highlight that many of the DEGs upregulated at 6 hpt were related to the SA pathway and participated in: (i) signal transduction (*BCS1*); (ii) the SA biosynthesis (*CaM* and the transcription factor *WRKY41*); (iii) the systemic acquired resistance (SAR) (*PNP-A* and methyltransferases); and (iv) pathogenesis-related proteins (PR) (*PR3, PR4, PR9*, *PR11,* and *PR14*). Other DEGs were related to ABA pathway such as the genes responsible for the synthesis of secondary metabolites (*CYP.1, MLP,* and *DXPS*), to JA and Et pathways (*LOX, AOC, ACS,* and *ACO*), and others were involved in other functions such as cell wall biogenesis (cellulose synthase) and defense signal transduction (kinases) (Table 2).

**Table 2:** The most interesting differentially expressed genes (DEGs) in almond plants treated with **flg22-NH**_**2**_ at 6 hpt associated with defense pathways

Related pathway^a^	Code^b^	ID^c^	Log_2_ FC^d^	FDR^e^	Protein and function^f^	Reference
SA	*BCS1*	Prudu.08G097700	1.40	3.49E−05	Encodes a protein that is present in a homo-multimeric protein complex on the outer mitochondrial membrane which amplifies SA signaling	Zhang et al. 2014
	*CaM*	**Prudu.05G208300**	1.11	2.92E−04	Calmodulin binding protein-like. It is a key regulator for ICS1 induction and SA biosynthesis	Yang and Poovaiah 2002; Zhang et al. 2010
	*DAHP2*	Prudu.01G471900	1.05	3.84E−06	Class-II DAHP synthetase involved in the synthesis of chorismate which is a precursor of SA	Weaver and Herrmann 1997; Peng et al. 2021
	*DXPS*	**Prudu.06G206800**	1.70	3.92E−04	Deoxyxylulose-5-phosphate synthase. Part of the 2-C-methyl-D-erythritol 5-phosphate (MEP) pathway that provides precursors for isoprenoid biosynthesis, biogenic volatile organic compounds, and redox cofactors with defense-related functions	Wright et al. 2014; Abbas et al. 2017
	*OMT1*	Prudu.04G046600	1.19	8.22E−06	Methyltransferase which could be related to the production of methylated SA	Métraux 2013
	*PLA2A*	**Prudu.06G248400**	1.67	4.96E−09	Phospholipase A 2A. Related to the production of JA, reactive oxygen species (ROS), and alkaloid production in response to biological stresses	Wang 2001
	*PNP-A*	Prudu.07G215300	1.13	1.83E−04	Plant natriuretic peptide A, which is a systemically mobile molecule related to SAR	Lee et al. 2020
	*PR3*	**Prudu.03G002500**	1.85	8.22E−06	Chitinase family protein (**PR3**). Hydrolysis of glycosidic bonds in chitin	Sels et al. 2008
		Prudu.01G187100	1.33	9.36E−05		
		Prudu.06G326300	1.13	1.24E−03		
		Prudu.01G187200	1.07	2.69E−04		
	*PR9*	**Prudu.06G232300**	4.27	1.02E−07	Peroxidase superfamily protein (**PR9**). Responsible for regulating ROS production which can act as secondary messengers and have antibacterial activity	Linthorst and Van Loon 1991
		Prudu.06G277400	1.73	8.11E−05		
		Prudu.07G016900	1.33	4.15E−06		
		Prudu.03G110000	1.21	3.25E−03		
	*PR14*	Prudu.06G040900	1.80	1.70E−03	Lipid transfer protein, which has been described to have antibacterial activity, among others	Kader 1996
		Prudu.06G007700	1.43	4.50E−04		
	*RLK*	**Prudu.01G271800**	1.05	3.80E−06	Receptor lectin kinase deployed during pathogen-triggered immunity. Can recognize PAMPs and initiate SA signaling cascades	Sun et al. 2020
	*WRKY41*	Prudu.02G282800	1.74	5.74E−04	WRKY transcription factor	Eulgem et al. 2000
ABA	*CYP.1*	**Prudu.08G183800**	2.81	5.28E−06	Encodes a cytochrome P450 (family 82, subfamily G, polypeptide 1). Biosynthesis of flavonoids and catabolism of isoprenoid hormones related to defense responses. Related to abiotic stress response	Xu et al. 2015; Pandian et al. 2020
	*CYP.2*	**Prudu.01G275100**	2.04	7.08E−08	Encodes a cytochrome P450 (family 72, subfamily A, polypeptide 9). Biosynthesis of flavonoids and catabolism of isoprenoid hormones related to defense responses. It also has monooxygenase activity. Related to abiotic stress response	Xu et al. 2015; Pandian et al. 2020
	*GST*	**Prudu.08G197200**	2.79	7.08E−08	Glutathione S-transferase. Detoxifies the plant from hyperperoxides. Functions in hormone transport and ISR. Related to abiotic stresses	Dixon et al. 2002; Gullner et al. 2018
	*MLP.1*	**Prudu.01G110600**	1.97	4.03E−10	MLP-like protein 423. Related to abiotic stresses mediated by ABA	Liu et al. 2020
	*MLP.2*	**Prudu.01G111200**	1.43	6.74E−06	MLP-like protein 423. Related to abiotic stresses mediated by ABA	Liu et al. 2020
	*NAC042*	Prudu.04G092900	2.43	4.18E−04	NAC domain containing protein 42 which is a transcription factor induced by H_2_O_2_ production and enhances tolerance to abiotic stresses	Zhang et al. 2022
JA	*AOC*	Prudu.03G217000	1.03	1.23E−03	Allene oxide cyclase which is related to JA biosynthesis	Ziegler et al. 2000
	*LOX*	Prudu.04G041300	3.90	3.57E−12	Lipoxygenases which are related to JA biosynthesis	Bannenberg et al. 2009
		Prudu.95S000400	1.08	7.23E−05		
Et	*ACO*	Prudu.03G194900	1.04	1.70E−08	1-Aminocyclopropane-1-carboxylic acid oxidase which is related to Et biosynthesis	Houben and Van de Poel 2019
	*ACS*	Prudu.05G109600	1.31	5.74E−04	1-Aminocyclopropane-1-carboxylic acid oxidase synthase related to Et biosynthesis	Houben and Van de Poel 2019
	*NAD(P)H*	**Prudu.01G233200**	1.52	6.21E−07	NAD(P)-linked oxidoreductase superfamily protein regulated by the Et pathway	Sellés Vidal et al. 2018
Other	*BCB*	Prudu.07G224600	1.26	2.51E−03	Blue-copper-binding protein that regulates the lignin biosynthetic process	Ji et al. 2015
	*CESA*	Prudu.01G020500	2.04	1.03E−03	Cellulose synthase related with cell wall biogenesis	Li et al. 2016
	*DNAJ*	**Prudu.03G092900**	1.47	4.55E−06	Chaperone DnaJ-domain superfamily protein. Protects antioxidant enzymes activity. Related with development processes	Fan et al. 2017; Wang et al. 2019
	*MFS*	**Prudu.06G255800**	1.79	1.71E−06	Major facilitator superfamily protein. It has a peptide transporter activity	Drew et al. 2021
	*MYB26*	Prudu.01G382600	2.04	1.32E−08	Transcription factor MYB26 related with cell wall biogenesis	Yang et al. 2007
	*SE*	**Prudu.01G411200**	1.50	3.84E−06	Sulfite exporter TauE/SafE family protein. It is part of the Cul3A-RING E3 ubiquitin ligase complex	Zhang et al. 2021
	*na*	Prudu.05G225400	1.03	8.47E−03	GRAM-domain containing protein related with cell wall biogenesis	Tiwari et al. 2020
	*na*	Prudu.02G131400	1.79	2.92E−04	Kinases involved in signal transduction	Hirt 1997
		Prudu.07G173300	1.26	4.03E−04		
		Prudu.03G139700	1.07	3.85E−05		

^a^*SA* salicylic acid pathway, *ABA* abscisic acid pathway, *JA* jasmonic acid pathway, *Et* ethylene pathway

^b^Assigned abbreviations correspond mainly to abbreviation codes used for *Arabidopsis thaliana. na* indicates that those genes do not have a general abbreviation assigned

^c^GenBank accession number. Codes in bold correspond to the selected DEGs for RT-qPCR experiments in this study

^d^Binary logarithm of the fold change (FC) expression of each transcript

^e^False discovery rate (FDR) of each transcript

^f^Protein codified in each transcript

## Gene markers related to almond plant response to flg22-NH_2_ treatment

A total of 15 DEGs (12 defense-related and 3 non-defense-related genes) in response to **flg22-NH**_**2**_ treatment were selected according to their log_2_ FC (Table 2). Sequencing of the obtained amplicons for the selected genes using the designed primers yielded the expected sequences. After optimization of the primer concentration for all of the selected genes, 300 nM was chosen as the final concentration for qPCR reactions. Standard curves of the 15 DEGs showed R-squared values above 0.99 and efficiencies above 80%, which allows the use of the ΔΔ*C*_*T*_ relative gene expression quantification method (Supplementary Table 2). No significant differences were observed between the endogenous genes *UBQ* and *TEF2* (*p* < 0.01), so *TEF2* was selected as a reference gene for relative gene expression quantification using the ΔΔ*C*_*T*_ method. A high correlation between the RNA-seq analysis results and the expression levels of the 15 DEGs was obtained through RT-qPCR. This result was confirmed by Pearson’s correlation test with a coefficient value of 0.92 (*p* < 0.01) (Supplementary Fig. S4).

## Effect of the application system of flg22-NH_2_ in almond plants and time-course analysis

**Fig. 2:**
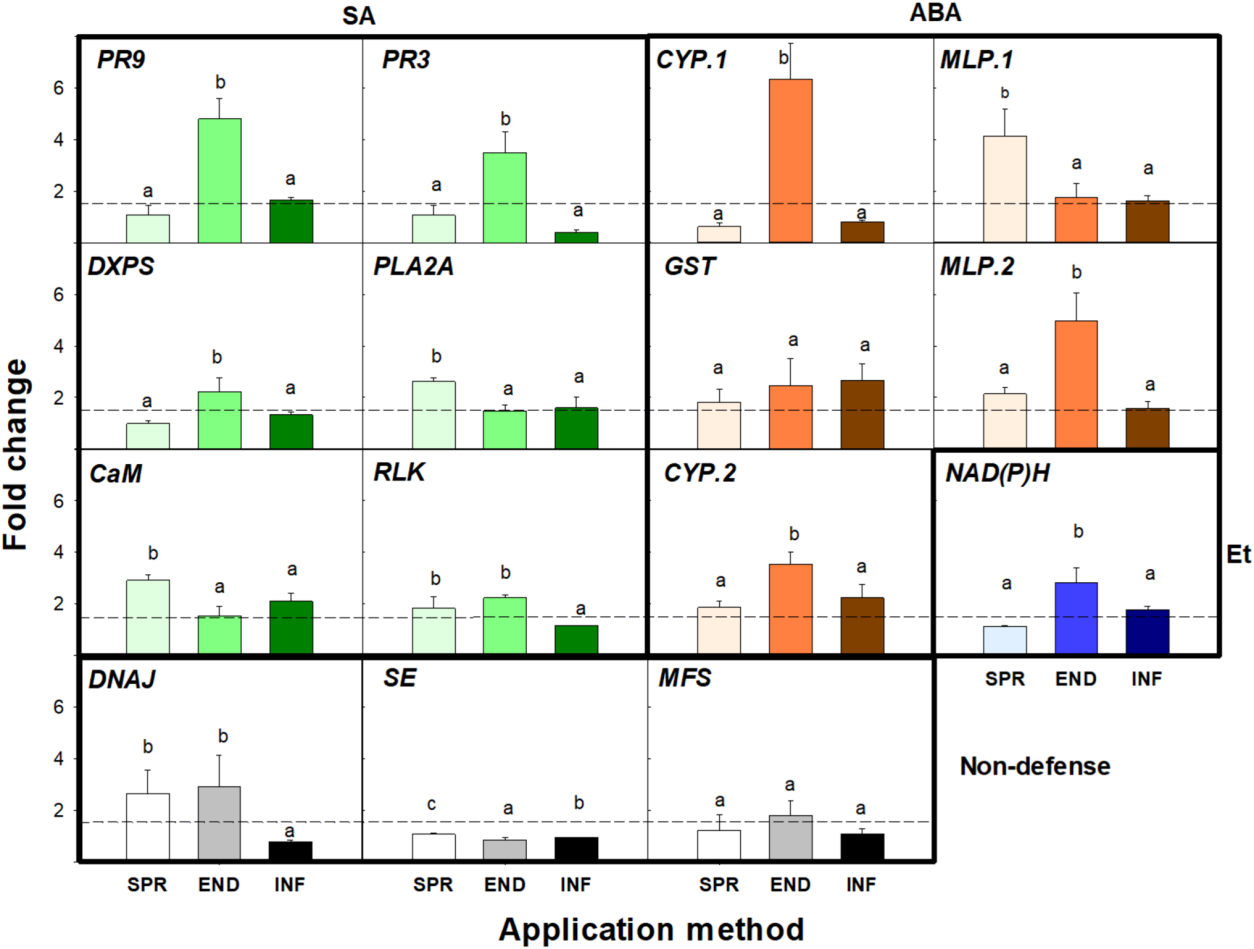
Effect of the application system (*SPR* spray, *END* endotherapy, *INF* infiltration) of **flg22-NH**_**2**_ in almond plants in the relative expression of selected **flg22-NH**_**2**_ responsive marker genes, classified into defense (greenish for SA, orangish for ABA, bluish for Et) and non-defense (grayscale) pathways at 6 h post-treatment. Genes were considered to be upregulated when they showed significant differences between their respective NTC (*p* < 0.05) and their fold change was higher than 1.5 (dashed line). Values are the means of three replicates of three plants each, and error bars represent the confidence interval (α = 0.05). Letters correspond to the means comparison between the different application systems for each gene. Means sharing the same letters within the same gene are not significantly different according to the Duncan’s test (*p* < 0.05) (colour figure online)

Three different strategies for peptide application on almond plants were evaluated to study their effect on gene expression: endotherapy, spray, and infiltration. The application of **flg22-NH**_**2**_ by endotherapy caused the upregulation of most of the selected genes (13 out of 15 genes), whereas spray and infiltration caused the upregulation of 8 out of 15 genes (Fig. 2**, **Supplementary Table S2), with infiltration being the worst strategy. Interestingly, the application strategy also influenced the intensity of gene upregulation. In particular, endotherapy led to the highest fold change in 7 out of 15 genes (*PR9*, *PR3¸DXPS, CYP.1, MLP.2, CYP.2, NAD(P)H*). The genes that showed the highest fold change when the peptide was applied by endotherapy were selected as markers for the following experiments (Supplementary Table S2).

**Fig. 3:**
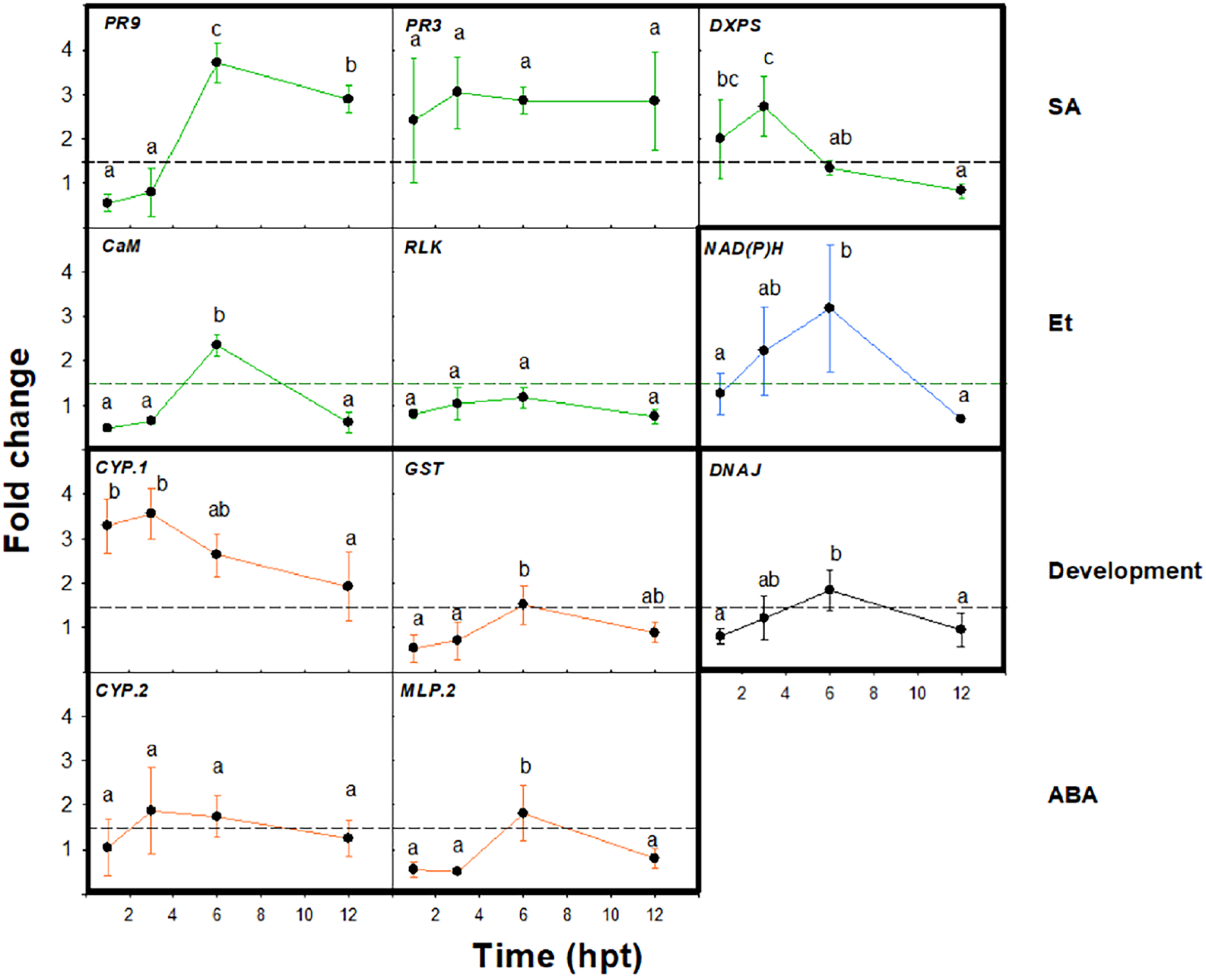
Time-course expression analysis of 11 selected genes of almond plants at 1, 3, 6 and 12 h after the treatment with **flg22-NH**_**2**_ by endotherapy. Genes classified into defense (green lines for SA, orange lines for ABA, blue lines for Et) and non-defense (black lines) pathways. Genes were considered to be upregulated when they showed significant differences between their respective NTC (*p* < 0.05) and their fold change was higher than 1.5 (dashed line). Values are the means of three replicates of three plants each, and error bars represent the confidence interval (*α* = 0.05). Letters correspond to the means comparison between the different sampling times for each gene. Means sharing the same letters within the same gene are not significantly different according to the Duncan’s test (*p* < 0.05) (colour figure online)

The time-course expression analysis of 11 selected genes of almond plants treated with **flg22-NH**_**2**_ by endotherapy was studied at 1, 3, 6, and 12 hpt. Three different expression patterns of DEGs were observed (Fig. 3). While *PR3*, *RLK,* and *CYP.2* presented a stable expression during the time-course experiment, *PR9*, *CaM*, *NAD(P)H*, *GST*, *MLP.2*, and *DNAJ* showed the highest fold change values at 6 hpt, and *DXPS* and *CYP.1* at 3 hpt. Therefore, the best sampling time to analyze the effect of **flg22-NH**_**2**_ on the plant defense response is at 6 hpt.

## Identification of new peptides with plant defense elicitor activity in almond plants

**Fig. 4:**
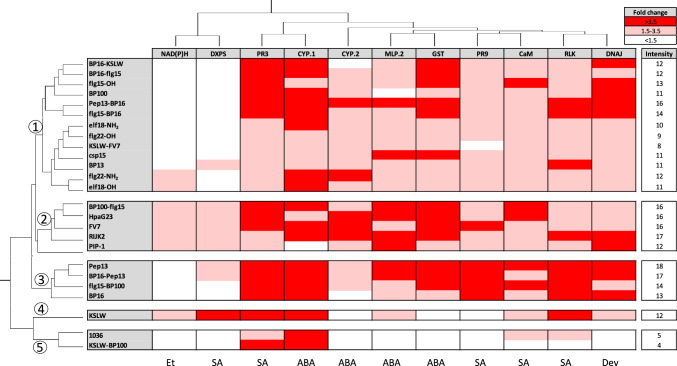
Heat map of the expression pattern of marker genes in almond plants after 6 h of the treatment with different peptides applied by endotherapy at 20 μM. Rows correspond to peptides and columns correspond to genes. The order of the peptides and the genes was established after hierarchical clustering using the Euclidean distance. Genes are colored depending on the intensity of upregulation caused by the treatment with the indicated peptide. Values are the means of three replicates of three plants each. Intensity of expression is represented as a numeric value that corresponds to the sum of the intensity of upregulation which for each gene is 0 when < 1.5-fold change, 1 when 1.5–3.5 and 2 when > 3.5

Almond plants were treated with the 25 peptides described above and their effect on the expression of the 11 genes previously selected was evaluated (Fig. 4**, **Supplementary Tables S2 and S7). Peptides induced different gene expression profiles that could be clustered into five groups related with their transcriptomic pattern. **BP100-flg15**, **HpaG23**, **FV7**, **RIJK2,** and **PIP-1** (group 2) and **Pep13**, **BP16-Pep13**, **flg15-BP100,** and **BP16** (group 3) led to a higher gene expression than the peptides of other groups. Interestingly, group 2 led to the upregulation of all genes and exhibited an expression intensity ranging from 12 to 17. Group 3 displayed an expression intensity ranging from 13 to 18, and had a high overexpression of the genes *PR3, CYP.1, GST, PR9,* and *RLK*. Peptides **BP16-KSLW**, **BP16-flg15**, **flg15-OH**, **BP100**, **Pep13-BP16**, **flg15-BP16**, **elf18-NH**_**2**_, **flg22-OH**, **KSLW-FV7**, **csp15**, **BP13**, **elf18-OH,** and **flg22-NH**_**2**_ (group 1) exhibited an expression intensity ranging from 8 to 16. Among them, **BP16-KSLW**, **BP100**, **Pep13-BP16,** and **flg15-BP16** induced a high upregulation of at least four genes, such as *PR3, CYP.1, GST,* and *DNAJ*. **KSLW** (group 4) exhibited an expression intensity of 12 and caused a high upregulation of *DXPS*, *PR3*, *CYP.1,* and *RLK*. Finally, **1036** and **KSLW-BP100** (group 5) only caused a high upregulation of *PR3* and *CYP.1* and displayed an expression intensity ranging from 4 to 5.

## Effect of peptide treatment on population levels of *X. fastidiosa* in almond plants

**Fig. 5:**
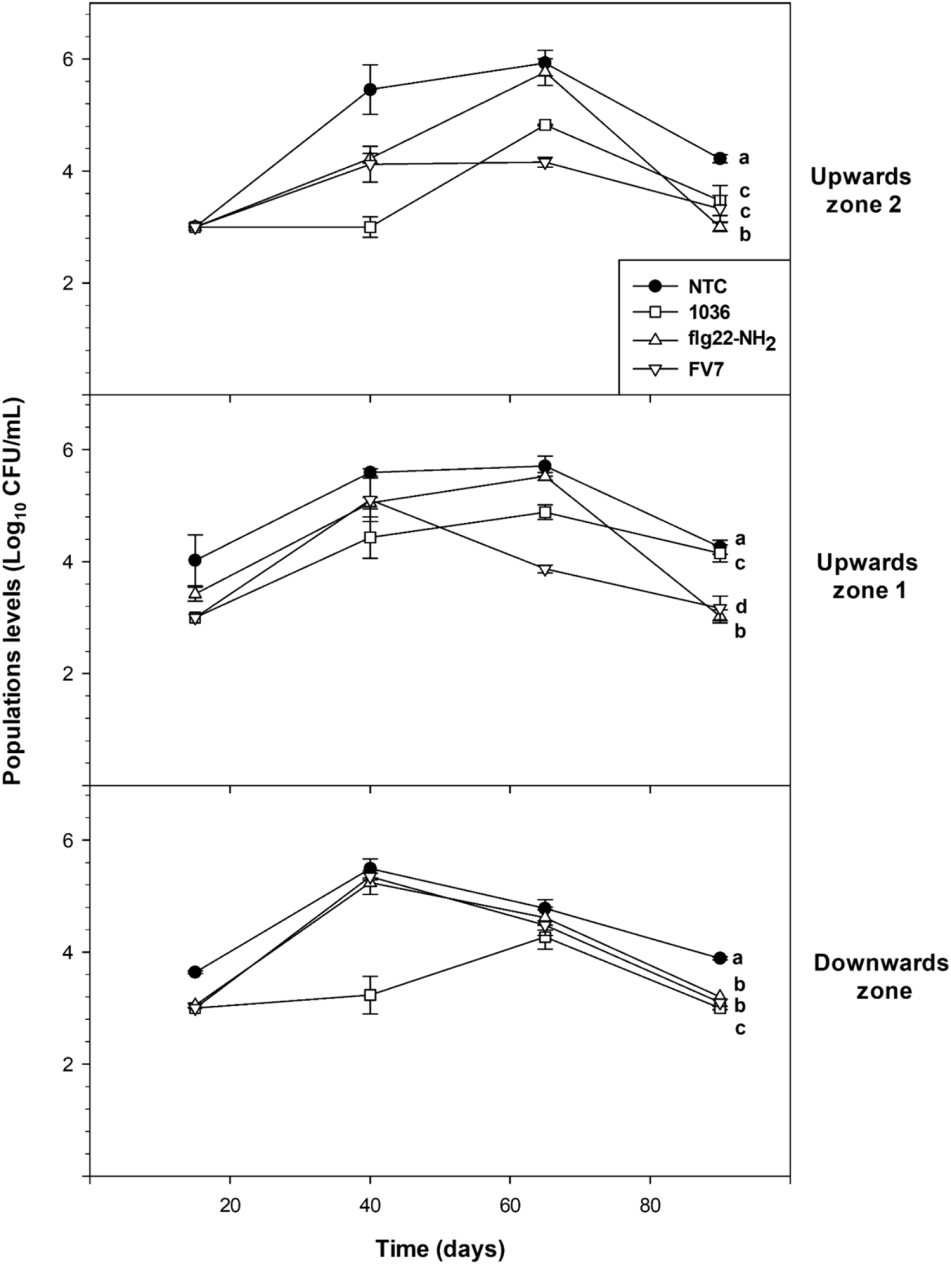
Effect of the treatments (**1036**, **flg22-NH**_**2**_ or **FV7**) on *X. fastidiosa* viable population levels in the sap of almond plants at 15, 40, 65, and 90 days post-inoculation (dpi). Values are the means of three biological replicates of three plants each, and error bars represent the confidence interval (*α* = 0.05). Different letters between treatments indicate significant overall differences between the treatments for each analyzed parameter according to Duncan’s test (*p* < 0.05)

No significant differences were observed between the results obtained from the two independent experiments at 40 dpi (*p* = 0.41) after treatment with the peptides **flg22-NH**_**2**_**, FV7,** and **1036**. The population levels of *X. fastidiosa* at 15, 40, 65, and 90 dpi are depicted in Fig. 5**.** All treatments showed overall significant differences through a repeated measures ANOVA in all of the plant sections that were analyzed (*p* < 0.05) when compared to the NTC. The most effective treatments were **FV7** and **1036,** which caused a significant reduction of the population of *Xff* in two of the three analyzed zones. Specifically, **FV7** caused the highest significant reduction of *Xff* viable cells in the upward zone 1 and upward zone 2 compared to the NTC, while **1036** caused it in the upward zone 2 and downward zone. The strongest reduction of viable *X. fastidiosa* cells in sap was higher than 2 log when compared with NTC in some of the analyzed times.

## ALS symptom development and leaf physiological parameter progression in treated almond plants

**Fig. 6:**
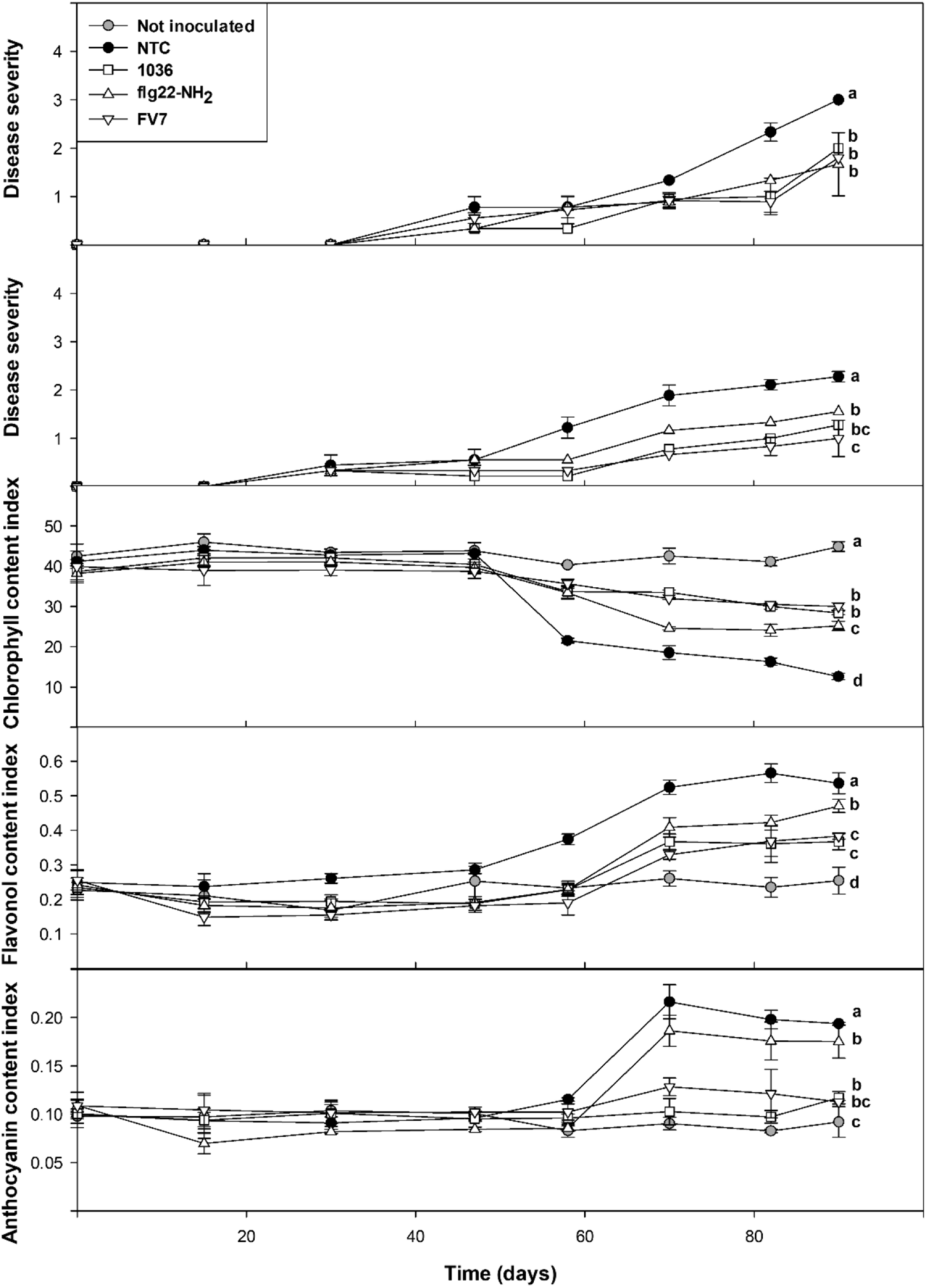
Disease severity and leaf physiological parameters (chlorophyll, flavonol and anthocyanin content index) of almond leaf scorch in plants inoculated with *X. fastidiosa* and treated with **1036**, **flg22-NH**_**2**_ or **FV7** by endotherapy compared to a not treated control (NTC) and a not inoculated control over a period of 90 days post-inoculation (dpi). For disease severity, values are the means of 9 plants divided in three biological replicates and the results of two independent experiments are shown. For the leaf physiological parameters, values are the means of four leaves/plant of a total of three biological replicates of three plants each and the results of one of the experiments are shown. Error bars represent the confidence interval (*α* = 0.05). Different letters between treatments indicate significant overall differences between the treatments for each analyzed parameter according to Duncan’s test (*p* < 0.05)

The disease severity and the progression of leaf physiological parameters (chlorophyll, flavonol, and anthocyanin content) were evaluated over a period of 90 dpi (Fig. 6 and Supplementary Fig. S5). ALS symptoms started between 30 and 47 dpi, and disease severity increased over time. NTC plants were the most affected during the whole experiment and most of them started to show marginal necrosis in almost half of the leaves at 90 dpi. In plants treated with the peptides **1036, flg22-NH**_**2**_, or **FV7**, ALS symptoms were reduced and displayed significant differences compared to the NTC plants throughout the two experiments. In particular, in the first experiment they showed 43%–62% of disease severity reduction at 82 dpi compared to the NTC (37%–61% reduction in the second experiment). No overall significant differences were found between **1036, flg22-NH**_**2**_, and **FV7** treatments in the first experiment, while in the second experiment, **FV7** caused a significant reduction in disease severity compared to **flg22-NH**_**2**_**.**

As expected, the NTC showed differences regarding the leaf physiological parameters when compared to the not inoculated control defining the maximum and minimum values for each parameter (lower chlorophyll and higher flavonol and anthocyanin content). Specifically, the leaf physiological parameter progression of inoculated plants can be divided in two phases, with the first one from 0 to 47 dpi and the second one from 47 to 90 dpi. During the first phase, the values of the parameters for the NTC and treated plants were similar which correlated with low disease symptoms. In the second phase, disease increased resulting in lower chlorophyll levels and higher flavonol and anthocyanin levels in all cases. Treatment with **1036** and **FV7** caused an increase in chlorophyll (85 and 88%) and a reduction of flavonol (35 and 36%) and anthocyanin content (51 and 39%) at 82 dpi when compared to the NTC.

Interestingly, when comparing the disease severity data with the corresponding leaf physiological parameters, a strong correlation was observed. Specifically, as assessed by Pearson’s correlation test, a coefficient value of −0.94 was obtained for chlorophyll content, 0.90 for flavonol content and 0.84 for anthocyanin content (*p* < 0.01).

## Discussion

*Xylella fastidiosa* is a plant pathogen which poses a great threat to the agricultural economy of the Mediterranean region, with almond being one of the most affected crops. Up to now, no strategy to completely cure infected plants has been found (Rapicavoli et al. 2018a; Sánchez et al. 2019; Saponari et al. 2019; Gibin et al. 2023). In the present work, we have identified peptides able to elicit defense responses of almond plants and demonstrated their capacity to control ALS caused by *X. fastidiosa*.

**Fig. 7:**
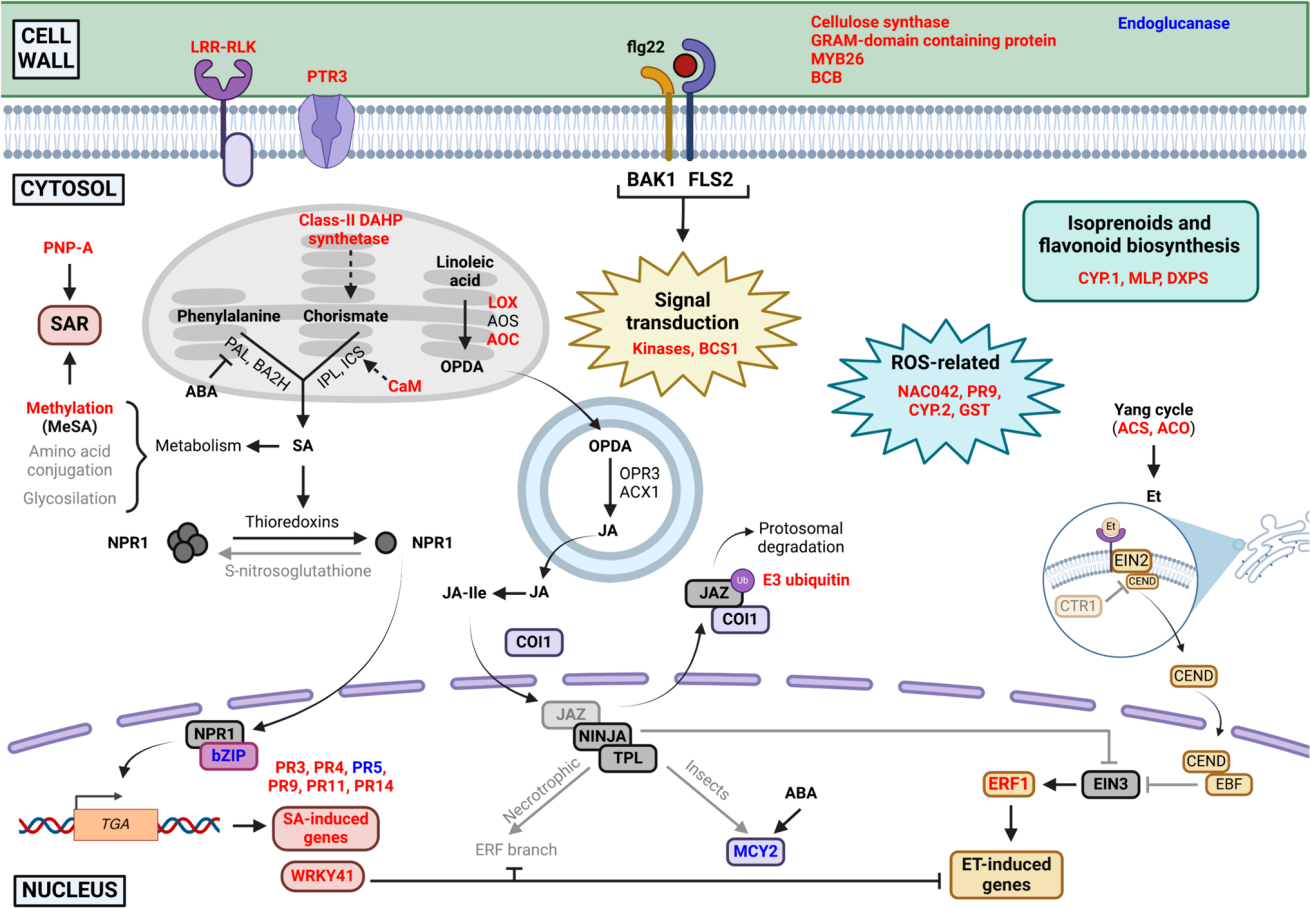
Defense related DEGs of *P. dulcis* after the treatment with **flg22-NH**_**2**_ at 6 h represented on the major plant defense pathways model. Red names correspond to upregulated genes and blue names to downregulated genes according to our study. Names in black correspond to proteins that are participating in the pathway. The codes of the proteins correspond to *A. thaliana* for easier interpretation*.* Image created with BioRender (https://biorender.com)

We report here the elicitor activity of the peptide **flg22-NH**_**2**_ in *P. dulcis* plants, since most of the identified DEGs by RNA-seq were related to the plant defense response. Although some DEGs were only upregulated at 6 hpt, showing a more transient expression, other genes related to plant defense such as *PR9* (*Prudu.06G232300*), *PR14* (*Prudu.06G040900*) and *LOX2* (*Prudu.95S000400*) were upregulated at both 6 and 24 hpt showing a more stable expression. The most relevant DEGs at 6 hpt (Table 2) are represented in a general model of the plant defense pathways (SA, JA, Et, and ABA) based on previous published studies (Fig. 7) (He et al. 2002; Liechti and Farmer 2002; Asselbergh et al. 2008; Sels et al. 2008; de Vleesschauwer et al. 2010; Derksen et al. 2013; Newman et al. 2013; Zhang et al. 2019; Ruan et al. 2019; Ali and Baek 2020; Lefevere et al. 2020; Malik et al. 2020). As depicted in Fig. 7, most of the upregulated genes were related to the SA pathway as described in other studies (van Verk et al. 2011; Mata-Pérez and Spoel 2019). Moreover, genes related to the JA and Et synthesis were also found to be upregulated, but the corresponding pathways were not completely activated, since relevant genes at the final steps, such as *MCY2,* were found to be downregulated or not detected. Generally, the activation of the SA pathway has been reported to result in the inhibition of the JA and Et pathway (Derksen et al. 2013; Altmann et al. 2020). However, it has to be taken into account that JA and Et not only have a role in the plant defense system, but seem to participate in other processes such as development and synthesis of secondary metabolites (Wasternack and Song 2016; Chang 2016) which would explain the upregulation of JA and Et biosynthetic genes in our study. Other upregulated genes related to ABA pathway were also identified, which are described to play significant roles in the plant defense system such as the synthesis of lignin and cellulose (Ton et al. 2009). In addition, the ABA pathway has also been described to integrate the signal networks of the SA, JA, and Et pathways and to modulate them (Asselbergh et al. 2008), which is aligned with the high number of ABA-related DEGs identified in this work.

In a previous study, we reported the plant defense elicitor activity of the bifunctional peptide **BP178** which exhibits antibacterial activity against bacterial pathogens, together with plant defense elicitor activity mainly mediated by the SA pathway (Moll et al. 2022). Similarities were observed when comparing the response of almond plants to the treatment with **BP178** and **flg22-NH**_**2**_. Remarkably, the transcriptomic profile was similar when comparing the response of almond plants to **BP178** at 24 hpt with that of **flg22-NH**_**2**_ at 6 hpt. At 24 hpt, both peptides shared 18 DEGs such as *thioredoxins, polygalactorunase inhibitors* (*PR6*; *Prudu.07G075200*), and *PR14* which are related to the SA pathway (Derksen et al. 2013). Nevertheless, **BP178** showed a higher number of upregulated genes than **flg22-NH**_**2**_. Interestingly, it has been previously demonstrated that **BP178** had a protective effect against *X. fastidiosa* infection (Moll et al. 2022), so it could be hypothesized that **flg22-NH**_**2**_ or peptides with a resembling plant defense activation mechanism would behave similarly. Nevertheless, since **BP178** is a bifunctional peptide and the focus of this work was to identify peptides with a single mechanism of action by acting as a plant defense elicitor, **flg22-NH**_**2**_ was chosen as a reference peptide in this work.

Taking into account that the strategy for peptide application may affect the plant response, different methods were tested in the present work. We demonstrated that endotherapy, consisting of an injection into the stem, is a better strategy than spray for the application of **flg22-NH**_**2**_ in *P. dulcis,* since it caused the upregulation of most of the selected genes*.* Considering that the plant defense response was studied in leaves, it is suggested that **flg22-NH**_**2**_, once introduced into the vascular system by endotherapy, could effectively reach its target site more efficiently than when applied by spray. Accordingly, a previous study demonstrated that **flg22-OH** was transported to distal parts when it interacted with its receptor FLS2 in *A. thaliana* (Jelenska et al. 2017). However, there are no studies describing the movement of peptides applied by endotherapy along the vascular system, so it should be studied in detail in the future. Additionally, endotherapy has other advantages compared to other application methods, since it allows precise administration and dosage of the active ingredient and avoids pesticide drift in agriculture applications, which result in a lower impact to the environment (Braekman et al. 2009; Ferreira et al. 2022; Grandi et al. 2023).

The analysis of selected gene expression at different times after treatment with **flg22-NH**_**2**_ allowed to study in more detail the time-course response of almond plants. Sampling times higher than 12 hpt were not used in the present study because most of the selected genes were not differentially expressed in the RNA-seq experiment at 24 hpt. Some genes such as *PR3* presented a stable overexpression throughout the study period, but other genes showed peak expression at early sampling times such as *CYP.1* and the remaining genes at later sampling times such as *PR9*. It is interesting to note that genes related to the synthesis of precursors such as *CYP.1* and *DXPS* were upregulated at early time points, specifically, between 1 and 3 hpt. This is in accordance with studies in *A. thaliana* where rapidly induced genes such as *CYP81F2* have peak expression at 30 min (Denoux et al. 2008). Other genes such as *PR9, GST,* and *DNAJ*, which are related to ROS, presented peak expression at 6 hpt. Similar results were observed in a study carried out with *Brachypodium distachyon* treated with **flg22** that presented the maximum number of DEGs at 6 h (Ogasahara et al. 2022).

The peptides tested in this study, including the reference peptide **flg22-NH**_**2**_, showed different expression patterns of the marker genes, being classified into five groups. The most interesting sequences were **HpaG23, Pep13, PIP-1, BP16, RIJK2, FV7, BP100-flg15, flg15-BP100,** and **BP16-Pep13** that caused a stronger plant defense response than **flg22-NH**_**2**_. **HpaG23, PIP-1,** and **Pep13** have been described as plant defense elicitor in *N. tabacum*, *S. lycopersicum,* and *Petroselinum crispum* (Nürnberger et al. 1994; Kim et al. 2004; Miyashita et al. 2011). Remarkably, in the present study, these peptides showed stronger elicitor activity than **flg22-NH**_**2**_ suggesting a heightened sensibility to those sequences in almond plants. In the case of **HpaG23**, its stronger activity might be explained, since it is a sequence obtained from *Xanthomonas* species which are causal agents of some diseases in almond (Weber et al. 2005; Wang et al. 2018). Other relevant peptides identified within this work were **BP16, FV7,** and **RIJK2**. Interestingly, **RIJK2** has been described to have other activities such as antibacterial and antibiofilm against *Xff* (Moll et al. 2021). Regarding **BP16** and **FV7,** they have been reported to display antibacterial activity against some Gram-negative bacteria, but not against *X. fastidiosa* (Badosa et al. 2007; De La Fuente-Núñez et al. 2012; Oliveras et al. 2022). In addition, in previous studies, we identified peptide conjugates with interesting results and observed that the monomers present in their sequence as well as the order of the conjugation have an important influence on their activity as plant defense elicitors (Oliveras et al. 2022). The most relevant peptide conjugates were **BP100-flg15, flg15-BP100,** and **BP16-Pep13**, resulting from the conjugation of **BP100** with **flg15** and **BP16** with **Pep13**. Peptide conjugates **BP100-flg15** and **flg15-BP100** displayed higher elicitor activity than both monomers, with the former displaying the highest elicitor activity. Regarding conjugates containing **BP16** and **Pep13**, the monomer **Pep13** and the conjugate **BP16-Pep13** exhibited the best activity.

Interestingly, some of the tested peptides were grouped with **flg22-NH**_**2**_ indicating that they induced a similar plant response in *P. dulcis*. Several previously described plant defense elicitors such as **elf18-OH** and **csp15** in *N. tabacum* and *A. thaliana*, respectively, fell within this group (Felix and Boller 2003; Kunze et al. 2004). This aligns with previous studies where the peptide **flg22-OH** and **elf18-OH** caused a similar defense response in *A. thaliana* (Aslam et al. 2009).

The protective effect of the plant defense elicitors **FV7** and **flg22-NH**_**2**_ against *Xff* infections was confirmed in almond plants. For all of the studied peptides, disease severity was reduced pointing out that the activation of the plant defense response by **FV7** and **flg22-NH**_**2**_ had a similar effect to that of a purely antibacterial compound such as **1036.** This protective effect of plant defense elicitors to fight diseases caused by *X. fastidiosa* has also been observed in grapevine, in which the application of lipopolysaccharides (LPS) of *X. fastidiosa* resulted in reduced Pierce’s disease symptoms (Rapicavoli et al. 2018b). Our results also align with a study that demonstrated that the application of the endophytic bacteria *Paraburkholderia phytofirmans* PsJN caused a reduction of disease severity by priming expression of innate disease-resistant pathways in grapevine (Baccari et al. 2019).

Disease severity in treated almond plants was significantly different from the NTC, but similar between treatments with the peptides. Nevertheless, differences could be appreciated in the leaf physiological parameters, with **FV7** and **1036** being the peptides with closer comparable values to the ones obtained for the not pathogen inoculated control plants. When comparing the inoculated and not treated control (NTC) with the not inoculated control, there were differences in chlorophyll, flavonol, and anthocyanin contents attributed to the pathogen infection, as previously described in the literature (Camino et al. 2021). It has been reported in *A. thaliana* and olive, orange, and almond trees that *X. fastidiosa* infection is characterized by a decrease in chlorophyll resulting in a detrimental effect on photosynthesis and in an increase in anthocyanin levels associated with the protective role of these compounds (Ribeiro et al. 2004; Purcino et al. 2007; Zarco-Tejada et al. 2018; Pereira et al. 2019; Camino et al. 2021). In the case of the peptide **1036**, these parameters were similar to those of the not inoculated control, probably due to its bactericidal activity as it can be observed in the decrease in viable *Xff* populations in sap at early sampling times which resulted in a delay in disease progression, similarly to that observed previously with the peptide **BP178** (Moll et al. 2022). Regarding **FV7**, since it does not have bactericidal activity, as far as we know its protective effect could only be attributed to its plant defense elicitor activity. The slight reduction in *X. fastidiosa* population could be attributed to the overexpression of *PR9* linked to ROS production which has antibacterial activity (Sels et al. 2008). Additionally, it could be related to genes such as *CYP.2* and *DXPS* involved in the synthesis of isoprenoids that have defense-related functions. Therefore, this would indicate that **FV7** is able to induce a primed state in almond plants. Nevertheless, it should be considered that **FV7** might have other mechanisms different from the one considered in this study. Therefore, further in-depth studies should be performed to fully understand the peptide’s mechanism of action.

In conclusion, we demonstrated that peptide **flg22-NH**_**2**_ is a plant defense elicitor peptide in *P. dulcis*. This peptide caused the upregulation of several defense-related genes, mainly the ones found in the SA and ABA pathways. The detailed study of the plant response to **flg22-NH**_**2**_ allowed to identify several genes, which were used as markers for plant defense response. The application of **flg22-NH**_**2**_ by endotherapy and sampling time of 6 hpt caused the strongest plant defense response, resulting in the highest number of upregulated genes and with the highest fold change values. In addition, this study allowed the identification of new plant defense elicitors in *P. dulcis* such as **FV7,** which has a protective effect in almond against *X. fastidiosa* infections. Therefore, the use of plant defense elicitor peptides could be a potential tool to manage almond diseases such as ALS.

### Supplementary Information

Below is the link to the electronic supplementary material.

Supplementary file1 (PPTX 135 KB)

Supplementary file2 (PPTX 1430 KB)

Supplementary file3 (PPTX 176 KB)

Supplementary file4 (PPTX 320 KB)

Supplementary file5 (PPTX 11722 KB)

Supplementary file6 (XLSX 13 KB)

Supplementary file7 (XLSX 13 KB)

Supplementary file8 (XLSX 4627 KB)

Supplementary file9 (XLSX 4972 KB)

Supplementary file10 (XLSX 80 KB)

Supplementary file11 (XLSX 75 KB)

Supplementary file12 (XLSX 13 KB)

## Data Availability

Raw data underlying the results presented in this study are available upon request. The RNA-seq data has been deposited in the GEO-NCBI repository with the code number GSE259385.
