# Supplementary material for: Prunus dulcis response to novel defense elicitor peptides and control of Xylella fastidiosa infections
Source: Plant Cell Rep. 2024 Jul 8;43(8):190. doi: 10.1007/s00299-024-03276-x (PMC11231009; doi:10.1007/s00299-024-03276-x)
Supplement: Supplementary file 1 — Supplementary file1 (PPTX 135 KB) [file 299_2024_3276_MOESM1_ESM.pptx]

## Slide 1
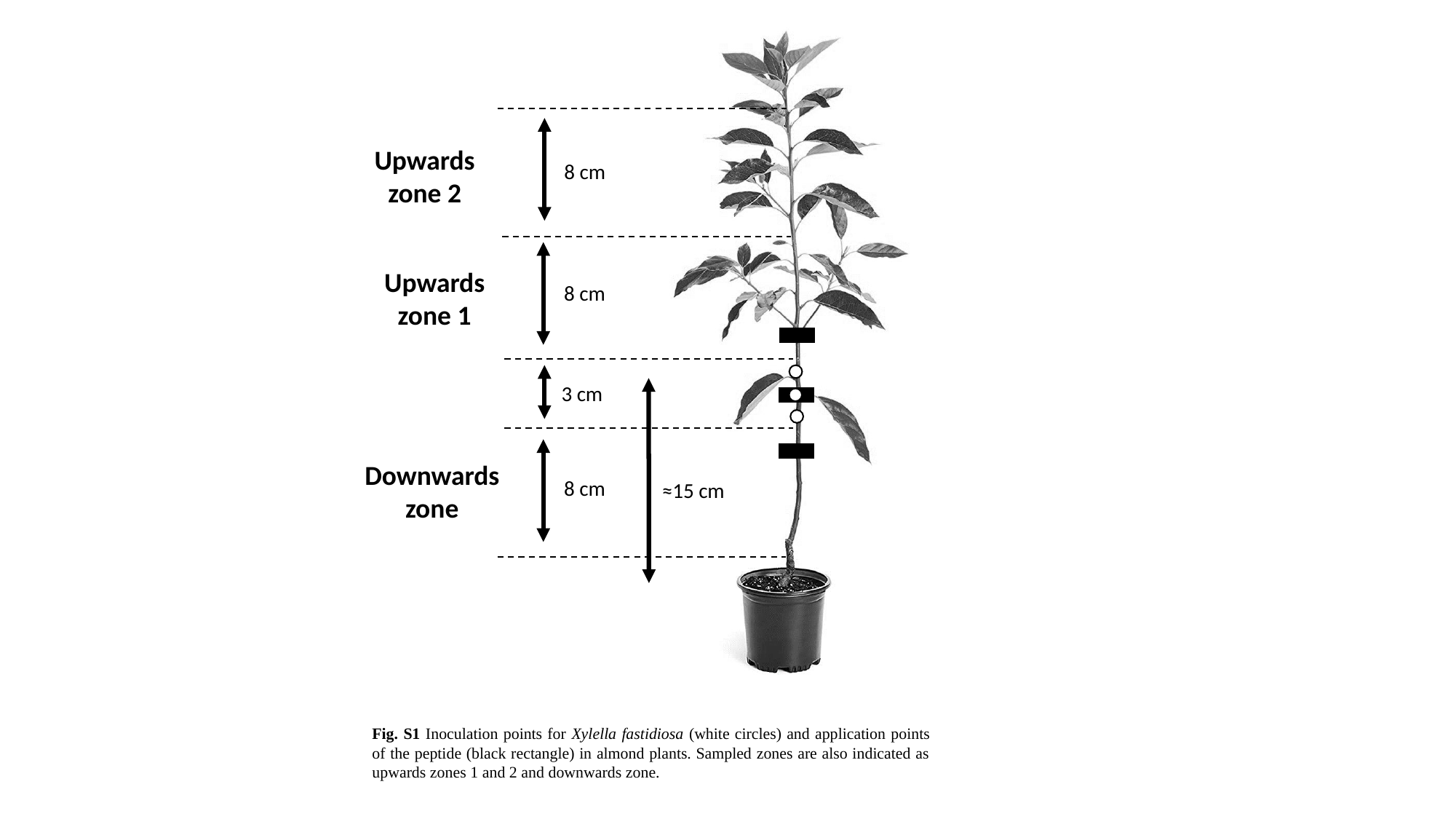

Upwards zone 2
8 cm
Upwards zone 1
8 cm
3 cm
Downwards zone
8 cm
≈15 cm
Fig. S1 Inoculation points for Xylella fastidiosa (white circles) and application points of the peptide (black rectangle) in almond plants. Sampled zones are also indicated as upwards zones 1 and 2 and downwards zone.
