# Supplementary material for: Prunus dulcis response to novel defense elicitor peptides and control of Xylella fastidiosa infections
Source: Plant Cell Rep. 2024 Jul 8;43(8):190. doi: 10.1007/s00299-024-03276-x (PMC11231009; doi:10.1007/s00299-024-03276-x)
Supplement: Supplementary file 2 — Supplementary file2 (PPTX 1430 KB) [file 299_2024_3276_MOESM2_ESM.pptx]

## Slide 1
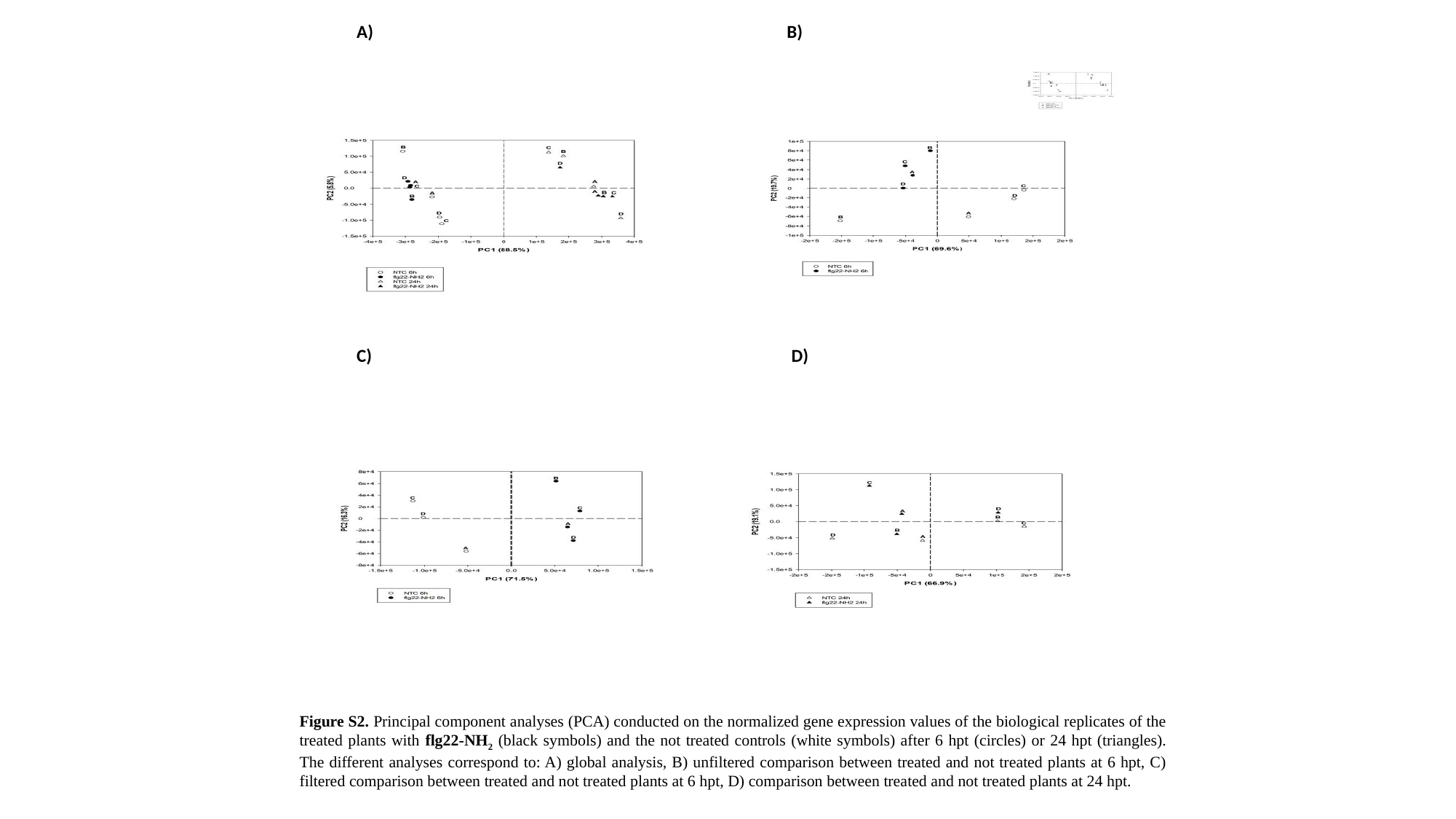

A)
B)
C)
D)
Figure S2. Principal component analyses (PCA) conducted on the normalized gene expression values of the biological replicates of the treated plants with flg22-NH2 (black symbols) and the not treated controls (white symbols) after 6 hpt (circles) or 24 hpt (triangles). The different analyses correspond to: A) global analysis, B) unfiltered comparison between treated and not treated plants at 6 hpt, C) filtered comparison between treated and not treated plants at 6 hpt, D) comparison between treated and not treated plants at 24 hpt.
