# Supplementary material for: Prunus dulcis response to novel defense elicitor peptides and control of Xylella fastidiosa infections
Source: Plant Cell Rep. 2024 Jul 8;43(8):190. doi: 10.1007/s00299-024-03276-x (PMC11231009; doi:10.1007/s00299-024-03276-x)
Supplement: Supplementary file 3 — Supplementary file3 (PPTX 176 KB) [file 299_2024_3276_MOESM3_ESM.pptx]

## Slide 1
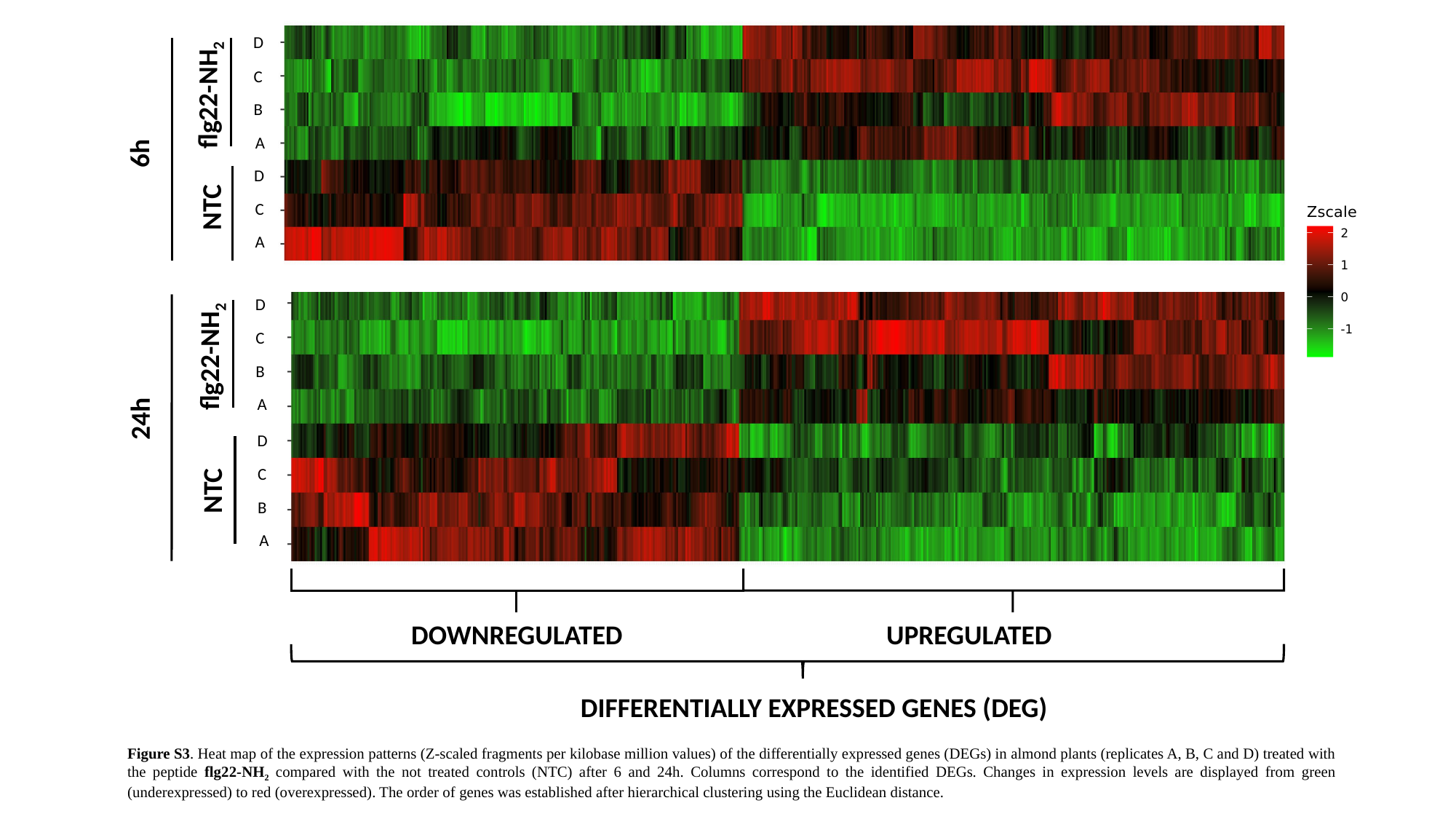

D
C
flg22-NH2
B
A
6h
D
NTC
C
A
D
C
flg22-NH2
B
A
24h
D
C
NTC
B
A
DOWNREGULATED
UPREGULATED
DIFFERENTIALLY EXPRESSED GENES (DEG)
Figure S3. Heat map of the expression patterns (Z-scaled fragments per kilobase million values) of the differentially expressed genes (DEGs) in almond plants (replicates A, B, C and D) treated with the peptide flg22-NH2 compared with the not treated controls (NTC) after 6 and 24h. Columns correspond to the identified DEGs. Changes in expression levels are displayed from green (underexpressed) to red (overexpressed). The order of genes was established after hierarchical clustering using the Euclidean distance.
