# Supplementary material for: Prunus dulcis response to novel defense elicitor peptides and control of Xylella fastidiosa infections
Source: Plant Cell Rep. 2024 Jul 8;43(8):190. doi: 10.1007/s00299-024-03276-x (PMC11231009; doi:10.1007/s00299-024-03276-x)
Supplement: Supplementary file 4 — Supplementary file4 (PPTX 320 KB) [file 299_2024_3276_MOESM4_ESM.pptx]

## Slide 1
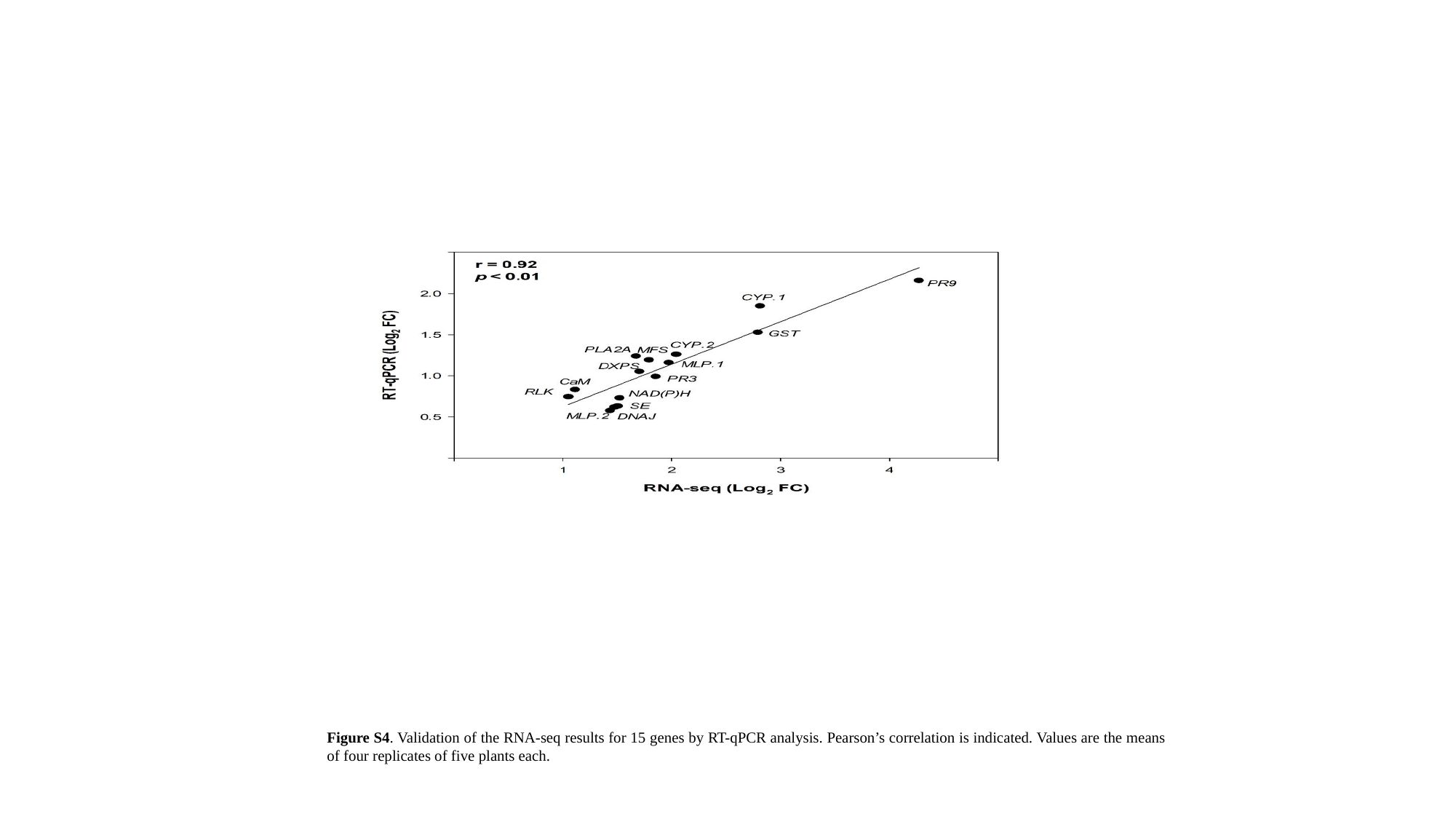

Figure S4. Validation of the RNA-seq results for 15 genes by RT-qPCR analysis. Pearson’s correlation is indicated. Values are the means of four replicates of five plants each.
