# Supplementary material for: Prunus dulcis response to novel defense elicitor peptides and control of Xylella fastidiosa infections
Source: Plant Cell Rep. 2024 Jul 8;43(8):190. doi: 10.1007/s00299-024-03276-x (PMC11231009; doi:10.1007/s00299-024-03276-x)
Supplement: Supplementary file 5 — Supplementary file5 (PPTX 11722 KB) [file 299_2024_3276_MOESM5_ESM.pptx]

## Slide 1
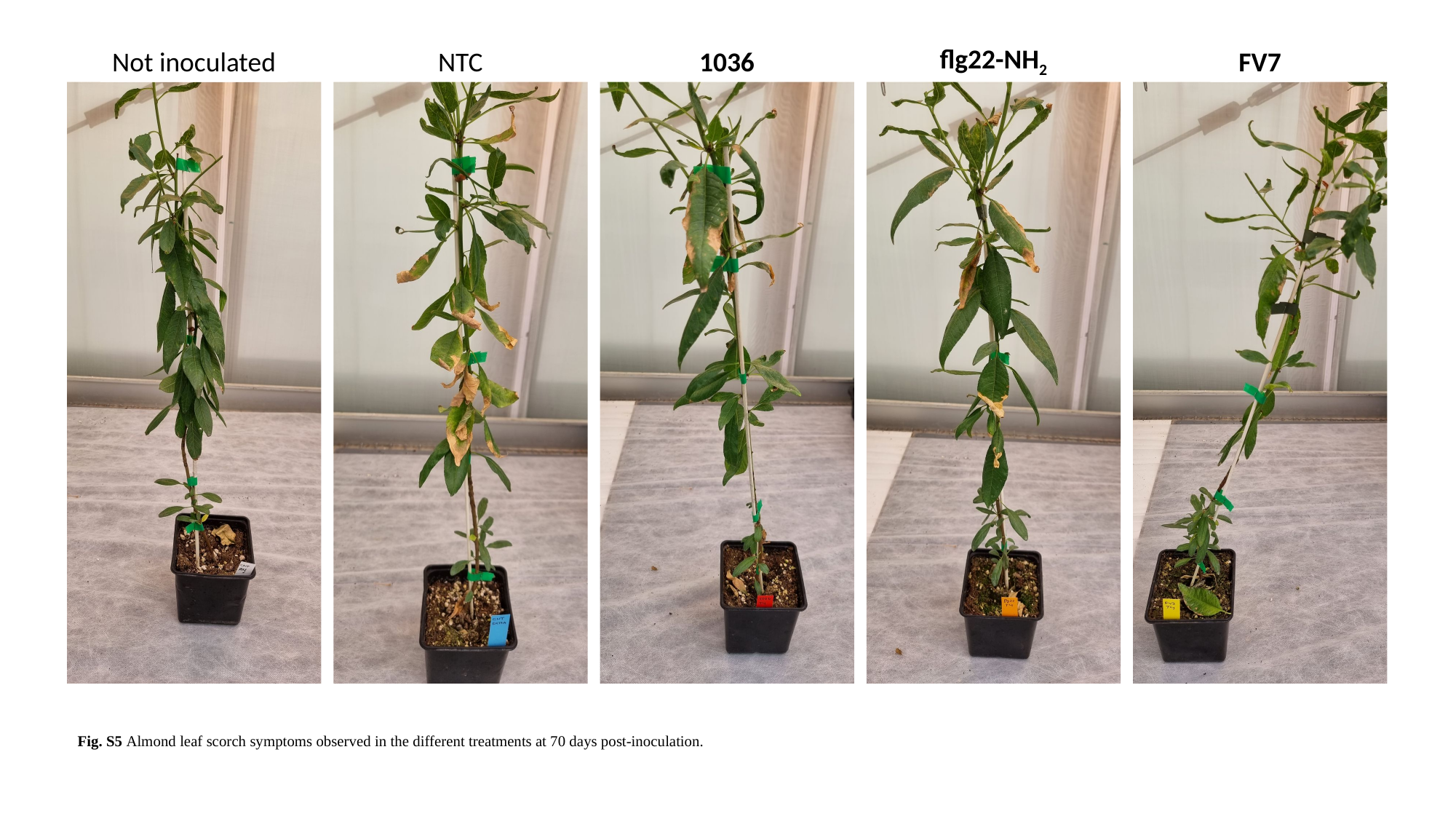

flg22-NH2
1036
NTC
Not inoculated
FV7
Fig. S5 Almond leaf scorch symptoms observed in the different treatments at 70 days post-inoculation.
